# Protein–DNA binding dynamics predict transcriptional response to nutrients in archaea

**DOI:** 10.1093/nar/gkt659

**Published:** 2013-07-26

**Authors:** Horia Todor, Kriti Sharma, Adrianne M. C. Pittman, Amy K. Schmid

**Affiliations:** ^1^Department of Biology, Duke University, Durham, NC 27708, USA and ^2^Center for Systems Biology, Institute for Genome Science and Policy, Duke University, Durham, NC 27708, USA

## Abstract

Organisms across all three domains of life use gene regulatory networks (GRNs) to integrate varied stimuli into coherent transcriptional responses to environmental pressures. However, inferring GRN topology and regulatory causality remains a central challenge in systems biology. Previous work characterized TrmB as a global metabolic transcription factor in archaeal extremophiles. However, it remains unclear how TrmB dynamically regulates its ∼100 metabolic enzyme-coding gene targets. Using a dynamic perturbation approach, we elucidate the topology of the TrmB metabolic GRN in the model archaeon *Halobacterium salinarum*. Clustering of dynamic gene expression patterns reveals that TrmB functions alone to regulate central metabolic enzyme-coding genes but cooperates with various regulators to control peripheral metabolic pathways. Using a dynamical model, we predict gene expression patterns for some TrmB-dependent promoters and infer secondary regulators for others. Our data suggest feed-forward gene regulatory topology for cobalamin biosynthesis. In contrast, purine biosynthesis appears to require TrmB-independent regulators. We conclude that TrmB is an important component for mediating metabolic modularity, integrating nutrient status and regulating gene expression dynamics alone and in concert with secondary regulators.

## INTRODUCTION

Diverse metabolic processes must be differentially regulated to maintain homeostasis and optimize growth in changing environmental and intracellular conditions. Environmental fluctuations occur at several temporal scales. This is mirrored in the integrated transcriptional and metabolic regulatory networks at the enzymatic, transcriptional and post-transcriptional levels in organisms responding to fluctuating conditions (1). For example, transient nutrient changes may result in microsecond-scale regulation of enzyme activity, whereas prolonged exposure during the course of minutes or hours may trigger changes in the levels of enzyme-coding transcripts (2). Because of the potential for the buildup of toxic intermediates and futile cycles, temporal dynamics of metabolic enzyme-coding transcripts can be as important as overall levels (3). Over evolutionary time scales, the constant presence of a given nutrient may lead to gene loss in competing metabolic pathways. Such streamlining of the genome is thought to enable faster replication and long-term adaptations in network structure or topology (4). Discerning the dynamic function of the underlying network resulting from the interaction of many different levels of regulation remains a central challenge.

In archaea, evidence is mounting that transcription may be an important mechanism for regulating metabolism. Unlike eukaryotes and bacteria, in which regulation of flux through central carbon metabolism and other pathways appears to be allosteric (5), archaea seem to lack many classic allosteric regulatory control points (6).For example, in hypersaline-adapted archaea, glutamate dehydrogenase was found to be unresponsive to ADP and GDP (7), whereas D-Lactate dehydrogenase was not regulated by fructose-1,6-bisphosphate (8). Additionally, pyruvate kinase from *Halobacterium salinarum* was found to possess only weak allosteric regulation (9). Other potential regulatory mechanisms such as protein phosphorylation have been proposed in two different archaeal species (10,11); however, in the hypersaline-adapted archaeal model system *H. salinarum,* much of the missing regulation seems to take place at the level of transcription (12–15). This property of haloarchaea provides a simplified model system for understanding the underlying logic of the integrated transcriptional and metabolic network. The largely transcriptional nature of archaeal responses has already been used to infer regulatory networks with a remarkable degree of accuracy (14). *H. salinarum* survives in an extreme and fluctuating environment, where daily and seasonal changes in salinity, oxygen and nutrients require constant adjustment of metabolism (16). Current evidence suggests that many of these metabolic adjustments are regulated transcriptionally.

For example, in *H. salinarum,* TrmB has been characterized as the central transcriptional regulator of carbon metabolic pathways under aerobic conditions (17). Conserved throughout the archaea, TrmB is a winged helix-turn-helix transcription factor (TF) that binds a palindromic inverted repeat *cis*-regulatory sequence in *Pyrococcus furiosus* (18)*, Thermococcus litoralis* (19) and *H. salinarum* (17). TrmB acts as a repressor at some promoters and as an activator at others*.* In the obligately anaerobic hyperthermophillic archaea, TrmB has been characterized as a regulator specifically involved in oligosaccharide transport and catabolism (20). In contrast, TrmB in *H. salinarum* has been found to bind 113 promoters in the genome to regulate genes involved in diverse processes including central carbon metabolism, TCA cycle, amino acid metabolism and co-factor metabolism (17).

Yet even in these simplified systems, where metabolic flux should largely be predictable from transcriptional data alone, regulatory causality is complicated by metabolic feedback. For example, TrmB appears to be regulated at the level of TF activity (14,17), binding sugars (glucose, trehalose, maltose) with varying affinities, which in turn decrease TrmB affinity for DNA (17–19). However, by de-activating transcription of *ppsA* (encoding phospho*enol*pyruvate synthase) and other gluconeogenic genes while de-repressing transcription of *pykA* (encoding pyruvate kinase) and other glycolytic genes, the amount of glucose is decreased, causing increased TrmB-promoter binding and reversing the effect (17). In bacteria, the enzymes involved in glycolysis and gluconeogenesis are regulated allosterically (5) in response to transient changes, and transcriptionally through cAMP Responsive protein (Crp in gram negative bacteria) in response to more permanent changes (21). In contrast, we hypothesize that TrmB regulates metabolic enzyme-coding genes rapidly in response to stimuli while also adapting the equilibrium levels of these genes to longer-term conditions.

Regulation of diverse pathways in response to environmental fluctuations of varying temporal scales is also likely to be mediated by several TFs working together in various combinations to produce appropriate transcriptional dynamics for each pathway (14,22). Such network topology is difficult to infer on the basis of steady state measurements of gene expression and promoter occupancy. To unravel cause and effect relationships in the gene regulatory network (GRN) controlling metabolism, we measured gene expression using NanoString® (23) and TrmB binding dynamics using ChIP-qPCR over time in response to a glucose stimulus. Integration of these data in the context of a dynamical model enabled prediction of how TrmB and its partners enact several transcriptional programs in different ways across metabolic pathways.

## MATERIALS AND METHODS

### Strains and growth conditions

*H. salinarum* NRC-1 (ATCC strain 700922) was used as the wild-type strain background for all studies (Supplementary Table S1). Gene expression was assayed in a previously constructed strain containing an in-frame deletion of VNG1451C [Δ*ura3ΔtrmB;* (17)] and its isogenic parent strain (Δ*ura3*). A previously constructed strain containing *trmB::c-myc* fusion on a low copy number plasmid was used for ChIP-qPCR to determine binding site occupancy (17). Cells for gene expression were grown in Complete Defined Medium containing 19 amino acids [modified from (17); Supplementary Table S2] supplemented with 50 µg/ml uracil to complement the *ura3* deletion. Strains carrying the *trmB::c-myc* construct for ChIP-qPCR were grown in Complete Defined Medium supplemented with 20 µg/ml mevinolin for plasmid maintenance. Cultures were grown at 225 r.p.m. shaking at 42°C under low ambient light.

### mRNA preparation for gene expression time course

*H. salinarum Δura3* (parent) and Δ*ura3ΔtrmB* (knockout mutant) strains were grown to early logarithmic phase (OD600 ≈0.3). For gene expression time courses, three 4 ml of aliquots were removed from the continuously shaking cultures before the addition of 5% (w/v) glucose (−240, −60, 0 min time points) and seven afterwards (5, 10, 20, 45, 90, 180, 360 min time points). Cells were immediately pelleted by centrifugation and snap frozen in liquid nitrogen. RNA was prepared using the Absolutely-RNA miniprep kit (Stratagene/Agilent) according to the manufacturer’s instructions. RNA quality was assessed using the Agilent 2100 BioAnalyzer RNA-Nano Chip and the Prokaryotic Nano RNA protocol (Agilent Technologies, Santa Clara, CA), and samples were verified to be free of DNA contamination by 25 cycles of PCR amplification on at least 200 ng of RNA before cDNA conversion in reverse transcriptase quantitative PCR (RT-qPCR).

### RT-qPCR

RNA extracted from cells was quantified using RT-qPCR. Briefly, RNA was quantified using the *Power* SYBR RNA-to-C_T_ 1-step kit (Applied Biosciences) in an Eppendorf Mastercycler ep Realplex thermocycler using Realplex software. Reaction size was 20 µl, and reactions were prepared according to manufacturer’s instructions. Plate setup was performed robotically using a Corbitt Life Sciences liquid handling system. C_Q_ threshold was determined automatically by the software. Primers were crosschecked against the *H. salinarum* genome using BLAST to ensure specificity. Amplification efficiencies for each pair of primers (*ppsA –* VNG0330G*, pykA –* VNG0324G*, eif1a2* - VNG1756G) were determined by running three serial 10-fold dilutions of the same sample (see Supplementary Table S3 for primers). These efficiencies were used to calculate enrichment relative to the reference gene (*VNG1756G*) in RNA from time course sampling from the measured C_Q_ values as previously described (24). Each experiment consisted of two to three biological replicates (separate cultures) and three technical replicates. The significance of change in gene expression before and after nutrient addition was assessed using the Welch one-sided *t*-test. Using the first three and last two points of each biological replicate time course, we determined *P*-values for the difference in means equivalent to a 1.5-fold up- or downregulation.

### Measurement of gene expression using NanoString

Quantification of additional genes was performed on the same RNA samples using NanoString technology (23). One hundred micrograms of each RNA sample from glucose time courses were delivered to NanoString (Seattle, WA), where samples were hybridized to a custom probe set (Supplementary Table S4) encompassing 100 genes of interest and counted using an nCounter machine. The same mRNA samples used for RT-qPCR were used for NanoString experiments. Data were normalized by total counts per sample across strains. All raw and normalized data are included in Supplementary Table S5. For clustering, each gene was further mean centered and normalized to a standard deviation of 1. Gene expression profiles were clustered using k-means with a Pearson correlation distance function on the normalized gene expression data with k = 8. The same clustering parameters were used for both Δ*ura3* parent and Δ*ura3ΔtrmB* mutant data sets. Each cluster was analyzed for enrichment of Clusters of Orthologous Groups (COG) categories (25,26) using the hypergeometric test.

### ChIP-qPCR protocol

*H. salinarum* cells harboring the *trmB::c-myc* construct were grown to early logarithmic phase (OD600 ≈0.3) as described earlier in the text. DNA–TF complexes were cross-linked and immunoprecipitated using the c-myc epitope tag as previously described (17). Primers were designed according to the criteria presented in (27). The qPCR thermocycling reaction and thermocycling conditions were as described previously (28), except that the SsoAdvanced SYBR Green Supermix (Bio-Rad) was used. Each of five biological replicates was run in three technical replicates. Enrichment of TrmB binding at the *ppsA* promoter was calculated as relative enrichment of the immunoprecipitated sample versus the input. The ratios given in the figures compare enrichment at the binding peak to the 3′ end of the gene of interest (28) (Supplementary Table S3).

### Degradation constant calculation

To assess the validity of our data and improve the accuracy of our modeling, we decided to incorporate genome wide mRNA degradation parameters. Although these parameters have been determined for a related strain [*H. salinarum* R1 ATCC 29341/DSM 671; (29)], the actual half-lives were not published. We therefore reanalyzed the microarray data (ArrayExpress accession number E-MEXP-1088, http://www.ebi.ac.uk/arrayexpress/) to determine these values (Supplementary Table S6). In short, we normalized each gene on each array to the t = 0 time point, and for each gene, fit an exponential decay curve through the points that were annotated as valid in the microarray file using the nls() function in the statistical package R. Standard error was also calculated using nls().

### Modeling approach and fitting

*Synthesis rate determination.* To better understand how binding events at the promoter influence gene expression, we calculated the derivative with respect to time for every gene at each time point by the weighted average of the slope of the segments before and after each time point. We used the K_deg_ values from (29) to calculate the synthesis rate according to Formula 1.
(1)
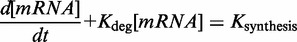



*Gene expression prediction*. To determine the importance of TrmB regulation to specific genes, we used an ordinary differential equation (ODE) model to predict gene expression dynamics in both WT and Δ*ura3ΔtrmB* strains as a function of ChIP-qPCR enrichment at the *ppsA* promoter. The first three and last three points of the *Δura3* gene expression and TrmB-binding time courses were used to determine the values for *K*_basal_ and *K*_eff_ by setting the right hand side of Formula 2 to 0. The Hill coefficient (n) was set to either negative or positive one to indicate repression or activation, respectively. The level of gene expression during the nutrient stimulus was predicted by calculating the change in gene expression every minute according to Formula 2.
(2)
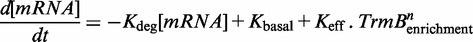



*Calculating model residuals.* To elucidate the topology of nutritional control of central and peripheral metabolism, we compared our predicted and actual gene expression data. Each gene was normalized to a maximum of 1. The normalized values were used as input to the TrmB-enrichment-based predictive ODE model of gene expression (earlier in the text). For each gene in each of the Δ*ura3ΔtrmB* and Δ*ura3* strain backgrounds at each mRNA time point, we calculated the difference between the predicted synthesis rate and the actual synthesis rate as calculated earlier in the text (see ‘Synthesis Rate Determination’ section). We clustered resulting traces on the basis of the Δ*ura3* data using k-means clustering with *k*=5 and Euclidean distance.

*Feed-forward loop logic approximation.* To assess whether a feed-forward loop (FFL) might be responsible for the dynamics we observed in the cobalamin biosynthesis cluster, we simulated the network using a logic approximation (Figure 8A) (30). Genes involved in cobalamin biosynthesis were normalized so that the lowest value was 0 and the highest value was 1. A regulator was presumed active when its value was >0.5. We used the TrmB ChIP-qPCR binding data as an input and fit the degradation parameter (*K*_deg FFL Effector_ & *K*_deg.cob/cbi_) for both of the other genes to the average scaled expression profile of the *cob/cbi* cluster using least squares. The fit was evaluated at 5, 10, 20 and 45 min following glucose addition. This is the period in which feed-forward dynamics would be expected to be most apparent.

## RESULTS

### Gene expression dynamics in response to nutrients are dependent on TrmB

**Figure 1.**
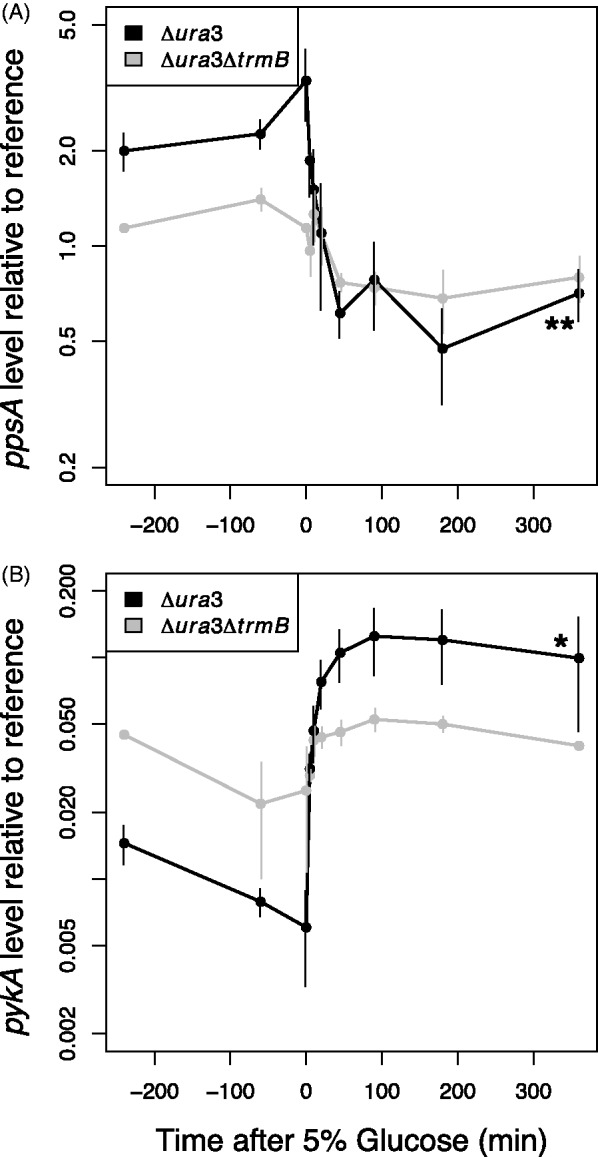
*ppsA* and *pykA* gene expression exhibits state-change dynamics in response to glucose perturbation when TrmB is present. Gene expression of *ppsA* (**A**) and *pykA* (**B**) to a 5% glucose stimulus in the Δ*ura3* (black lines) and Δ*ura3ΔtrmB* (gray lines) strains were measured by RT-qPCR and are shown plotted on a logarithmic axis. Error bars represent the standard error from the average of at least two biological replicate experiments. Asterisks indicate significance of the difference in expression level between the beginning and the end of the time course; * significant at *P* < 0.05; ** significant at *P* < 0.01; *** significant at *P* < 0.001.

**Figure 2.**
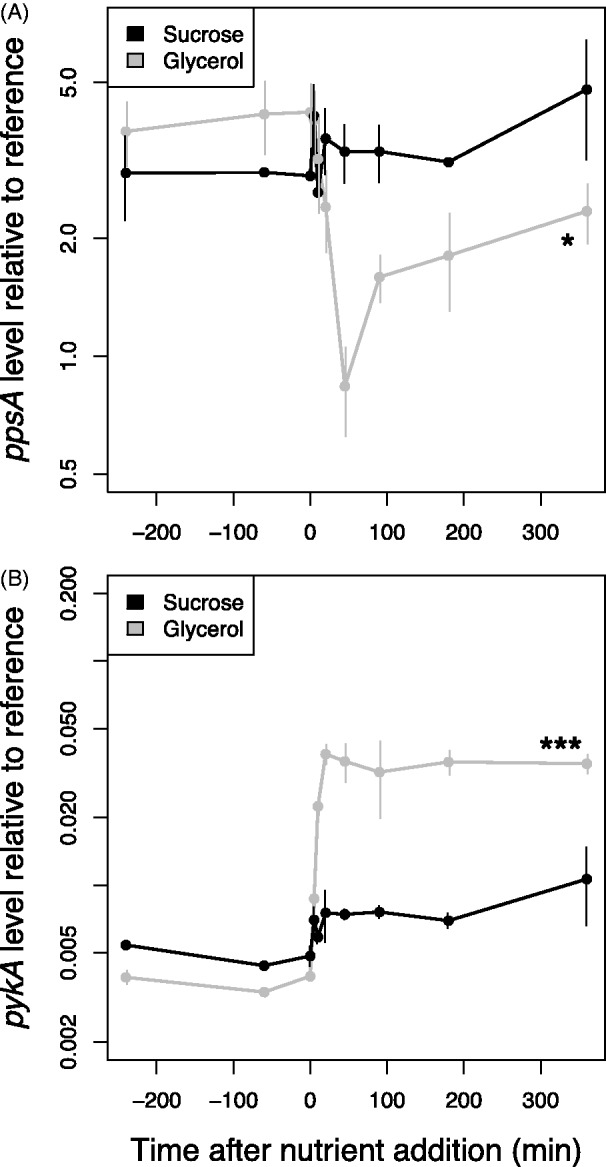
*ppsA* (**A**) and *pykA* (**B**) do not respond significantly to 5% sucrose in the Δ*ura3* strain (black lines). *ppsA* and *pykA* respond to 0.167% glycerol stimulus in the Δ*ura3* strain (gray lines). Gene expression was measured by RT-qPCR and is shown plotted on a logarithmic axis. Asterisks indicate significance of the difference in expression level between the beginning and the end of the time course; * significant at *P* < 0.05; ** significant at *P* < 0.01; *** significant at *P* < 0.001.

Our previous work demonstrated that TrmB binds the promoter of metabolic enzyme-coding genes throughout the genome in response to carbon source availability (17). However, these studies were conducted at steady state. To investigate the dynamic expression response of TrmB-regulated genes to nutrients (glucose, glycerol and sucrose), we used RT-qPCR to measure repressed (*pykA*) and activated (*ppsA*) gene levels in response to nutrients over time (Figures 1 and 2). TrmB is thought to regulate these genes by binding to the promoter either to activate or to repress expression in the absence of glucose. Addition of glucose to the medium results in TrmB dissociation from the promoter and de-activation or de-repression of the target gene (17). Briefly, *H. salinarum* cells were grown on amino acids as a carbon and energy source to mid-logarithmic phase. Cells were sampled thrice before and seven times after the addition of nutrients (‘Materials and Methods’ section). We considered a change relevant when a 1.5-fold or greater up- or downregulation of the target gene was significant (*P* < 0.05). As these genes are a key control point in glycolysis (*pykA*) and gluconeogenesis (*ppsA*), their levels are highly informative of the regulation of that pathway (31,32).

We observed that both *ppsA* and *pykA* mRNA exhibit a characteristic steady-state expression value during mid-logarithmic phase growth before glucose addition that differs between the Δ*ura3* parent and Δ*ura3ΔtrmB* mutant strain (Figure 1). On addition of glucose, *ppsA* is de-activated (Figure 1A, 3.6-fold decrease) and *pykA* is de-repressed (Figure 1B, 12.7-fold increase) by the first time point (5 min), and both genes reached a new equilibrium level by 45 min. Hereafter, we refer to such monotonic changes in gene expression as state-change dynamics. These dynamics were greatly attenuated in the Δ*ura3ΔtrmB* mutant background in response to glucose (Figure 1) and in the Δ*ura3* parent strain in response to sucrose (Figure 2). Gene expression dynamics similar to those in response to glucose were observed with glycerol (Figure 2), although ultimately, a different final equilibrium level was reached. These data confirm that gene expression dynamics of *ppsA* and *pykA* are specific to glucose and glycerol and dependent on TrmB. Furthermore, they confirm the role of TrmB in regulating genes coding for enzymes in glycolysis/gluconeogenesis at both short and intermediate temporal scales.

### NanoString measurement of temporal gene expression reveals metabolic modularity governed by TrmB

**Figure 3.**
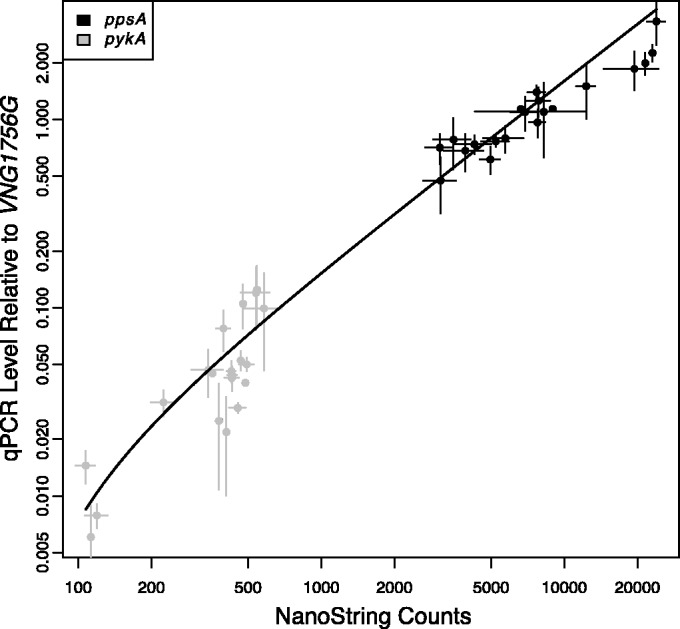
NanoString gene expression measurements are tightly correlated with qPCR measurements. mRNA levels for *ppsA* (black points) and *pykA* (gray points) were measured by RT-qPCR and NanoString. Both RT-qPCR and NanoString are shown plotted on a logarithmic axis. Error bars represent standard error from two replicates of NanoString and at least two replicates of RT-qPCR.

In our initial experiments, both *ppsA* and *pykA* gene expression exhibited state-change TrmB-dependent dynamics. Because these genes are an important control point in glycolysis/gluconeogenesis (6), state-change dynamics in response to glucose and glycerol were expected. However, TrmB regulates genes involved in processes across metabolism (17). To assess the impact of TrmB on gene expression in response to glucose, we assayed mRNA levels over time following glucose stimulus for 100 genes involved in central and secondary metabolism using NanoString (23). NanoString was used to assay gene expression because it has been shown to be sensitive, precise and require minimal processing (33). We selected 96 genes involved in central metabolism, including both direct and indirect TrmB targets and TrmB itself (Supplementary Table S4). Using data from previous microarray experiments (13,14,34,35), we also selected three genes (*VNG1670C, mrp, srp19*) expressed at high, medium and low levels with minimal variation across many conditions (Supplementary Table S4). The same glucose response time points used for RT-qPCR (Figure 1) were sampled in the NanoString experiment. As NanoString has not been previously used for mRNA quantification in *H. salinarum*, we validated the data by comparing measurements of *ppsA* and *pykA* gene expression in the same RNA time course samples between the NanoString and RT-qPCR platforms. We found a linear relationship (Pearson correlation = 0.962) across several orders of magnitude (Figure 3), suggesting that NanoString is a robust alternative to RT-qPCR for the quantification of mRNA in *H. salinarum*.

**Figure 4.**
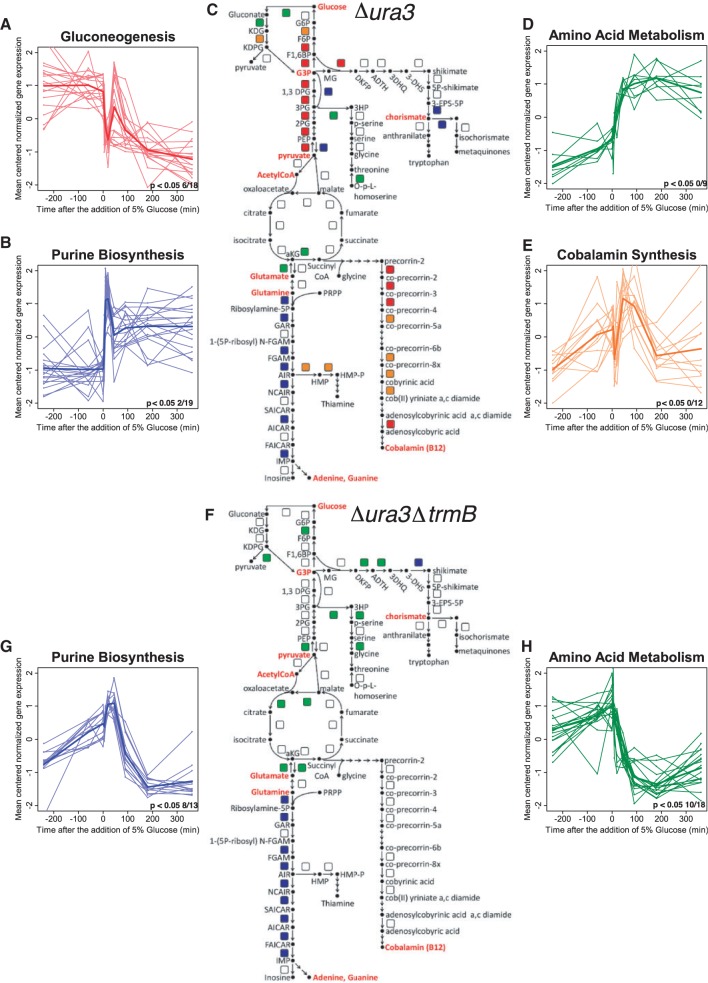
TrmB is required for metabolic modularity in *H. salinarum* metabolism. Figure depicts k-means clustering and using eight clusters and Pearson correlation as a scoring metrics on the Δ*ura3* (**C**) and Δ*ura3ΔtrmB* (**F**) data. Only clusters enriched (*P* < 0.01) for COG biological function categories are shown for each strain. Four clusters are enriched in genes involved in specific pathways in Δ*ura3* (**A**, **B**, **D** and **E**), whereas only two clusters are thus enriched in Δ*ura3ΔtrmB* (**G** and **H**)*.* Metabolic maps (C and F) depict enzyme-coding genes as colored squares, with colors corresponding to cluster graphs. Each cluster graph depicts gene expression data for individual genes (thinner lines) and the mean expression profile for the cluster (thicker lines). The number of genes in every cluster that shows significant (*P < *0.05) 1.5-fold over expression by the one-sided Welch *t*-test is listed in the lower right-hand corner of each expression graph. Metabolite abbreviations used in (C and F) are listed in Supplementary Table S7. In all, 30 of the 100 genes analyzed by NanoString are not represented on the metabolic map for the sake of clarity.

To determine the pattern of TrmB-dependent regulation across central and secondary metabolic pathways, NanoString gene expression profiles were clustered separately in the Δ*ura3* and Δ*ura3ΔtrmB* strain backgrounds using k-means. The distribution of clusters was integrated with the TrmB-centered metabolic regulatory network reconstruction (17) (Figure 4 and Supplementary Table S5). Clusters of genes from both *Δura3* and Δ*ura3ΔtrmB* expression profiles were analyzed for significant (*P* < 0.01) enrichment of membership in COGs (Table 1) (25, 26). Surprisingly, we found that temporal gene expression patterns clustered according to metabolic pathway modules in the *Δura3* strain and to a lesser extent in the Δ*ura3ΔtrmB* mutant, suggesting that TrmB is at least partially responsible for the maintenance of metabolic modularity (Figure 4C and F and Supplementary Table S5 and Table 1).

**Table 1.** *P*-values of enrichment of COG terms in k-means clusters of dynamic expression profiles of the Δ*ura3* and Δ*ura3ΔtrmB* strains after glucose stimulus

COG term	Description	*Δura3*	*Δura3ΔtrmB*
Carbohydrate transport and metabolism	Glycolysis/gluconeogenesis	1.08E-03	NS
Amino acid transport and metabolism		2.95E-03	7.32E-04
Nucleotide transport and metabolism	Purine biosynthesis	1.35E-04	1.23E-09
Coenzyme transport and metabolism	Cobalamin biosynthesis	4.47E-03	NS

NS = not significant.

The gluconeogenic enzyme-coding genes exhibited the same state-change downregulation dynamics as *ppsA* in the Δ*ura3* strain, with the exception of enolase (*eno*), which is not a direct TrmB target (17). These dynamics were not observed in the Δ*ura3ΔtrmB* strain (Figure 4A and Table 1). Because state-change dynamics in gluconeogenic enzyme-coding genes were greatly diminished in the Δ*ura3ΔtrmB* strain, it is likely that these genes are predominantly dependent on TrmB. As expected, the expression profiles of enzyme-coding genes in gluconeogenesis clustered in the Δ*ura3* parent strain but not in the Δ*ura3ΔtrmB* strain (Table 1).

In contrast to the state-change dynamics (*i.e.* monotonic increases or decreases and a statistically significant change in equilibrium gene expression) observed in the glycolytic and gluconeogenic pathways, impulse-like dynamics (transient increases or decreases in gene expression) were observed in the genes coding for enzymes in purine, cobalamin and amino acid biosynthesis in the *Δura3* strain. The variety of impulse-like dynamics made metabolic modularity especially apparent in these clusters. The pattern of gene expression in the purine biosynthesis cluster was distinct in both the Δ*ura3* and Δ*ura3ΔtrmB* strains (Figure 4B and G and Table 1). Expression of these genes displayed a transient increase in mRNA immediately on the addition of glucose in both backgrounds (Figure 4B and G). Although the impulse-like dynamics observed in purine biosynthesis genes are not TrmB-dependent, the difference in overall mRNA concentration compared with the parent strain at the start of the time course but not at the end suggests that TrmB is nonetheless important in controlling these genes.

Expression patterns in genes coding for enzymes involved in cobalamin (vitamin B-12) biosynthesis also differed between the Δ*ura3ΔtrmB* mutant and Δ*ura3* parent strains. In the Δ*ura3* parent strain, these genes were downregulated as soon as glucose was added. This was followed by a transient upregulation centered 45 min after the addition of glucose (Figure 4E). These distinct expression patterns were tightly correlated and clustered together in the *Δura3* strain (Figure 4E)*.* In contrast, in the Δ*ura3ΔtrmB* background, these genes were slowly and constantly upregulated following the addition of glucose without any rapid changes in expression level (Supplementary Figure S1). There was no significant clustering of these expression profiles in the Δ*ura3ΔtrmB* knockout (Table 1). Together, these data suggest that cobalamin biosynthesis is predominantly TrmB regulated but that other factors may be involved.

In summary, the surprising diversity of observed dynamic gene expression patterns suggests that TrmB is required for temporal coordination of gene expression across metabolism in response to glucose. These patterns implicate other unknown regulatory factors in this process. The expression of genes coding for enzymes at the core of central metabolism appears to be predominantly TrmB regulated, while branching cofactor pathways seem to require additional, as of yet unidentified regulators.

### TrmB promoter occupancy can explain gene expression dynamics for *ppsA*

**Figure 5.**
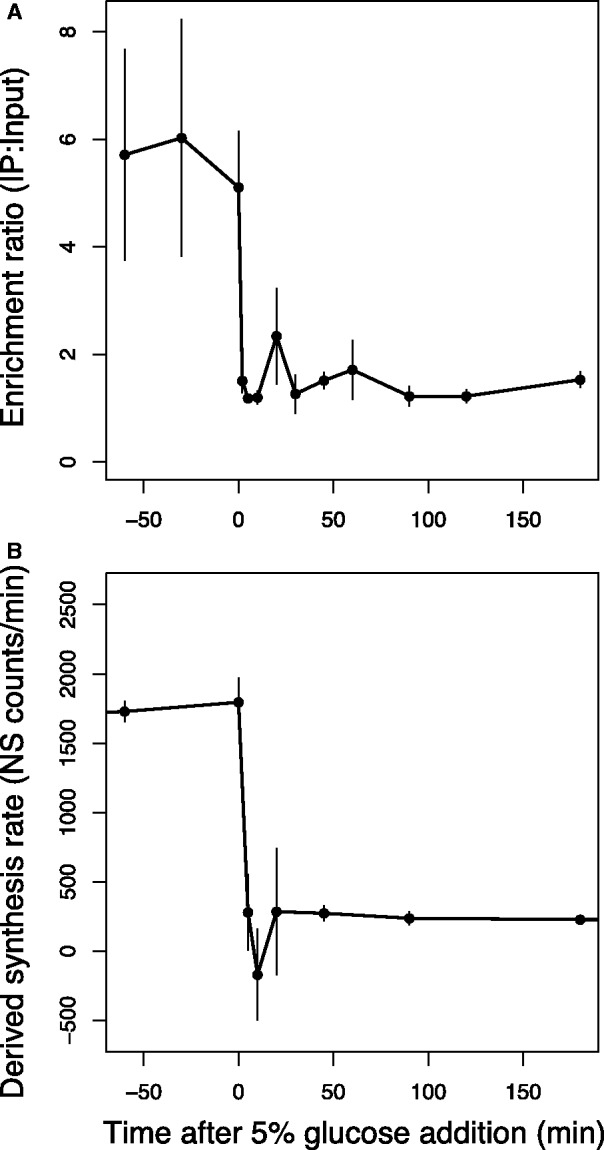
(**A**) TrmB-binding enrichment at the *ppsA* promoter is correlated with (**B**) predicted mRNA synthesis rate inferred from the gene expression data (‘Materials and Methods’ section) at the TrmB-dependent *ppsA* promoter after the addition of 5% glucose. Error bars represent the standard error from the average of biological replicate experiments. See also Supplementary Figure S3.

To determine the specific contribution of TrmB-promoter binding to gene expression dynamics, we assayed TrmB enrichment over time in response to glucose at the *ppsA* promoter using chromatin immunoprecipitation followed by qPCR (ChIP-qPCR, ‘Materials and Methods’ section). Binding was assayed from 60 min before glucose addition through 180 min after addition. The same time points used for gene expression measurement were sampled with five additional time points for increased resolution. During mid-logarithmic growth on amino acids as a sole source of carbon and energy, TrmB showed significant binding enrichment (Figure 5A). On the addition of glucose, TrmB dissociated from the *ppsA* promoter within 2 min, and binding dynamics exhibited slight but reproducible damped oscillations before reaching a new steady state level (Figure 5A). To quantitatively assess the role played by TrmB in the gene expression profile of *ppsA*, we calculated a derived mRNA synthesis rate and compared it with promoter occupancy. We reasoned that a derived mRNA synthesis rate is representative of actual promoter activity and deconvolves the effect of differing mRNA degradation rates in different genes.

Derivation of an mRNA synthesis rate from mRNA data requires the degradation constant (K_deg_) for the gene of interest. For this, we used genome-wide mRNA degradation rates that have been experimentally determined previously in *H. salinarum* (29). The derived mRNA synthesis rate of *ppsA* inferred from the gene expression data (Figure 5B) correlated strongly with the ChIP-qPCR measurement of actual enrichment (Pearson correlation = 0.94). Taken together, these calculations suggest that TrmB is the primary regulator of *ppsA* mRNA synthesis.

### Prediction of gene expression based on TrmB-promoter enrichment

**Figure 6.**
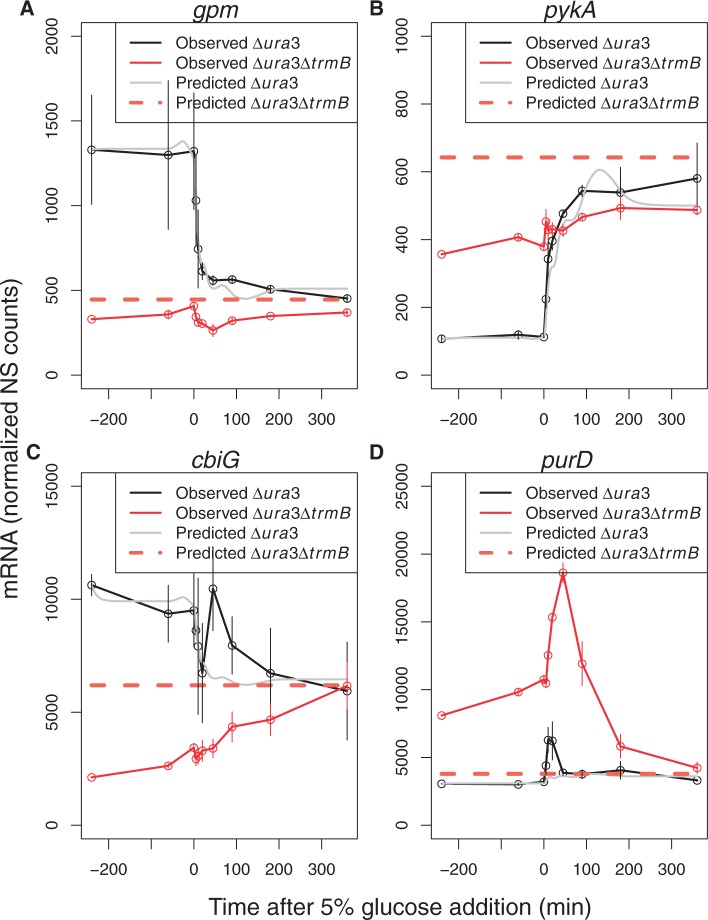
ODE model fit to NanoString gene expression data in response to glucose is predictive of gene expression in the *Δura3* (parent strain) and Δ*ura3ΔtrmB* mutant background for some but not all genes. (**A**) Phosphoglycerate mutase (*gpm*). (**B**) Pyruvate kinase (*pykA*). (**C**) Cobalamin biosynthesis gene (*cbiG*). (**D**) 5-phosphoribosylglycinamide (GAR) synthetase (*purD,* purine biosynthesis pathway). Black lines represent mRNA level in the *Δura3* strain. Red lines represent mRNA level in the *ΔtrmB* strain. The gray line and the dotted orange line show model fits for *Δura3* and Δ*ura3ΔtrmB,* respectively. Error bars depict standard error from the average of two biological replicates of the gene expression data. Model fits to data for the remaining 96 genes are exhibited in Supplementary Figure S1.

TrmB dynamics at the *ppsA* promoter explained the temporal gene expression pattern observed for *ppsA* mRNA (Figure 5). This led us to ask to what extent TrmB-promoter binding dynamics alone could explain the temporal expression patterns of other genes in the TrmB regulon. To address this question, we modeled expression across the 100 genes in the NanoString data set as a function of TrmB enrichment at the *ppsA* promoter over time. As TrmB binds a *cis*-regulatory motif that is conserved throughout its regulon (TACT-N_(7__–8)_-GAGTA) and its biochemical mechanism involves reduced affinity for DNA when sugar is present in the growth medium, we expect that TrmB binding will be qualitatively similar at all regulated promoters. This is supported by previous genome-wide ChIP-chip studies showing that TrmB bound its regulon only in the absence of glucose or glycerol (17). We therefore assumed for computational modeling purposes that TrmB dynamics at the *ppsA* promoter were representative of those at other promoters in the genome. *ppsA* promoter-binding dynamics (Figure 5) were used as an input to an ODE model of gene expression with two parameters: a basal synthesis rate (K_basal_) and a scaling term (K_eff_)(Methods). For 62 genes, this approach adequately explained the dynamics and predicted the level of gene expression in the Δ*ura3ΔtrmB* mutant strain (Figure 6A and B and Supplementary Figure S1). For other genes, this method explained certain aspects of the dynamics, but not all. For yet other genes, the model was unable to explain the changes in gene expression (Figure 6C and D). The modeling and prediction for each of these groups is described in turn later in the text.

**Figure 7.**
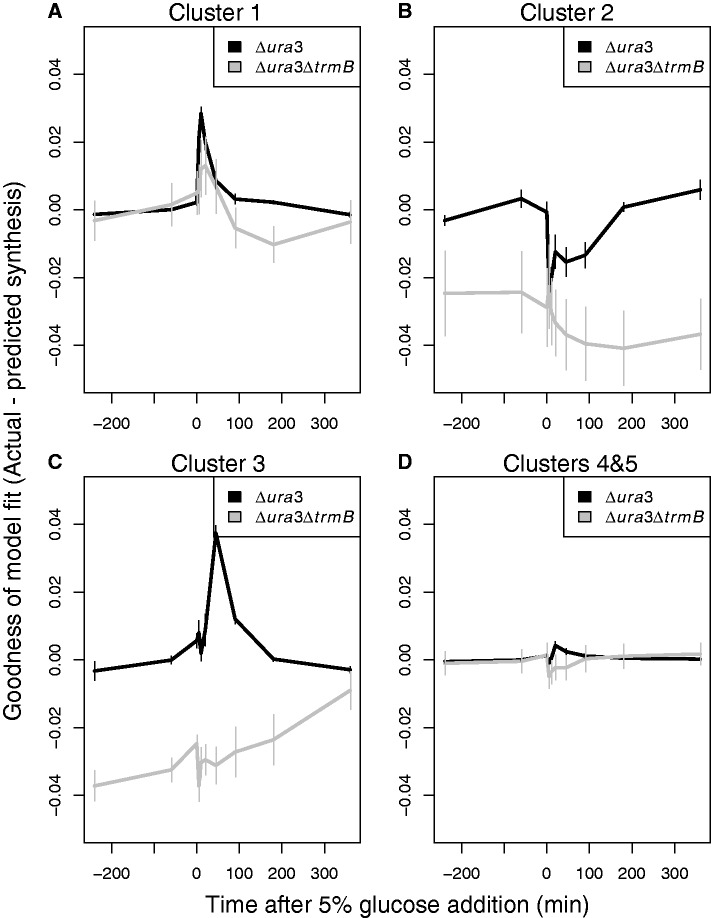
The difference between model-predicted and actual gene synthesis rates in Δ*ura3* (black) and Δ*ura3ΔtrmB* (gray) in clusters over time in response to glucose. (**A**) Cluster 1, enriched in genes coding for enzymes involved in purine biosynthesis; (**B**) Cluster 2, enriched in genes coding for enzymes involved in amino acid metabolism; (**C**) Cluster 3, enriched in genes coding for enzymes involved in cobalamin biosynthesis; (**D**) Clusters 4 and 5, containing genes whose expression is well-fitted by our model. The error bars represent the standard error from the average of the model fit residuals in each cluster.

To characterize both the role and the temporal dynamics of additional regulators working with TrmB over time in response to glucose, we calculated the difference in gene synthesis rate between the model prediction and the observed data. We then clustered the synthesis rate residuals (actual minus predicted) in the Δ*ura3* strain for each gene over the glucose response time course using k-means. This approach classified the accuracy of model fits to the data into five different groups (Figure 7). All five clusters were highly enriched for specific COG functions (Table 2). In two of the five clusters (Cluster 4 and 5, Figure 7D), containing 62 of 100 genes, no pattern was found in the residuals. This suggests that these genes exhibited either little change over the time course or that expression predictions from the TrmB-enrichment model were accurate (Figure 7D). As expected, all three of the no-change control genes were members of these two clusters. Further, carbohydrate metabolism genes were enriched in one of these clusters: they are predominantly TrmB regulated and were therefore accurately predicted (Figures 5 and 6).

**Table 2.** *P*-values of enrichment of COG terms in k-means clusters of Δ*ura3* model residuals

Cluster number	COG term	*P*-value
Cluster 1	Nucleotide transport and metabolism	6.30E-03
Cluster 2	Amino acid transport and metabolism	3.19E-02
Cluster 3	Coenzyme transport and metabolism	1.39E-06
Cluster 4	Carbohydrate transport and metabolism	7.24E-03
Cluster 5	Energy production and conversion	4.73E-02

The three remaining clusters (Clusters 1, 2, 3; Figure 7 A–C) showed significant dynamic patterns in their model residuals between 0 and 180 min after perturbation with glucose. This indicates that the model prediction deviated significantly from the observed data over certain time points. Further, this deviation suggested that other regulators besides TrmB may be involved.

### Inferring regulatory network topology from dynamic gene expression output

To identify how such regulators may be involved, we reversed biological circuit design principles (2) to infer GRN structure from dynamical output. Patterns of regulation that could not be predicted from TrmB-promoter-binding data were analyzed using logic approximations. We applied this to Clusters 1 and 3, which were enriched for functions in purine and vitamin B-12 biosynthesis, respectively (Figure 7 and Table 2).

**Figure 8.**
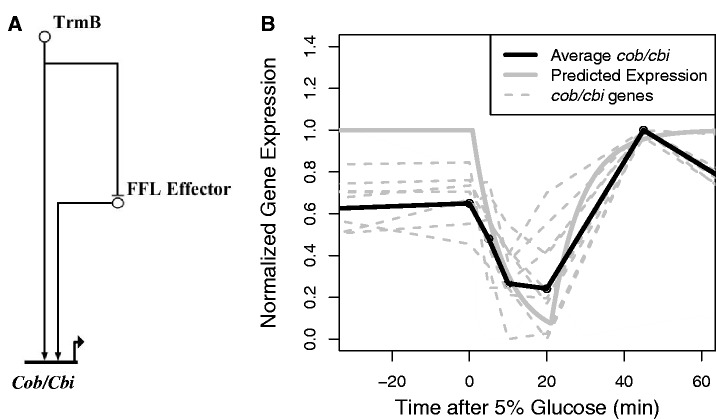
(**A**) An FFL is the most likely topology for the regulation of cobalamin synthesis. Model of proposed FFL involved in cobalamin biosynthesis is shown. (**B**) Fit of regulatory logic simulation with FFL topology and gene expression time course data. The thick gray line represents the best model fit to the *cob/cbi* average expression (black line). The dotted gray lines represent the scaled expression profiles of individual genes.

The Δ*ura3* residuals in Cluster 3 (enriched for genes encoding cobalamin biosynthesis proteins) exhibited a peak centered at 20–45 min (Figure 7C and Table 2). Consistent with the gene expression data, the spike was absent in the Δ*ura3ΔtrmB* strain. The global time delay from changes in transcript to changes in protein level following perturbation in *H. salinarum* has been estimated at ∼16 min based on parallel mRNA and proteomics time course data (13). The similarity between global transcription-translation time lag and the lag between glucose addition and the peak observed in cobalamin biosynthesis gene residuals at 20–45 min suggest that TrmB and a second TrmB-dependent regulator may be involved in a FFL to regulate cobalamin biosynthesis. As we observed an immediate decrease in gene expression after TrmB dissociated from the DNA, we reasoned that either TrmB or the FFL regulator could activate cobalamin gene expression. This represents an OR logic gate in the FFL. To test whether our data were consistent with such a feed-forward regulatory topology, we simulated the proposed network using logic approximations (30) (Figure 8A). We then fitted the parameters of the simulation to our cobalamin biosynthesis (*cob*/*cbi*) gene expression data over the first 60 min following glucose addition (‘Materials and Methods’ section). The fitted degradation rate of the FFL effector was 0.034 min^−^^1^. This indicates a response time of ∼20 min, which is consistent with the 16 min lag between transcription and translation (13), and supports the transcription-translation FFL hypothesis (Figure 8B). From this model, the degradation constant for the *cob*/*cbi* mRNA was estimated at ∼5.5 min, which is similar to the average empirically determined half-life of the *cob*/*cbi* mRNA [8.0 min; (29)]. Furthermore, the dynamic profiles of these genes make it clear that TrmB is acting as an activator of *cob/cbi* genes, and that the second regulator is acting as an activator as well.

In contrast to the lagged residuals of the cobalamin biosynthesis cluster, the residuals of Cluster 2, enriched in arginine and serine metabolism genes, exhibited an immediately changing dynamic residual profile in the Δ*ura3* parent strain (Figure 7B and Table 2). Model residuals increase immediately on glucose addition in the Δ*ura3* parent strain. This suggests that TrmB is not a primary regulator of these genes. In contrast to the previous cluster, because of the diversity of amino acid metabolic pathways and the likelihood of multiple independent regulators, further understanding of network topology of this cluster will require additional studies.

The residuals of Cluster 1, enriched for functions in purine biosynthesis (Figure 7A and Table 2), increased immediately following glucose addition. Furthermore, the residuals were similar in both Δ*ura3* and Δ*ura3ΔtrmB.* As the response time through a TF would be on the order of the half-life of this factor [∼10 min; (2)], these data implicate a secondary regulator already present in the cytoplasm. Although TrmB may ultimately control the concentration of such a secondary regulator (Supplementary Figure S2), initial dynamics are likely TrmB-independent. As the input signals to this secondary regulator are unknown, this network is not amenable to a logic simulation. Nevertheless, these data support the hypothesis that a secondary regulator may work through an independent pathway to impinge on the purine biosynthesis genes coordinately with TrmB (Figure 6D).

In summary, predictions from ODE and logic models suggest that TrmB is responsible, in various capacities, for the temporal regulation of metabolism as well as the overall gene expression levels. We conclude that integrating temporal gene expression data in response to environmental and genetic perturbation assists in the clarification of cause-effect relationships and prediction of the GRN topology that regulates metabolic pathways.

## DISCUSSION

In this study, we integrated dynamic gene expression data with ChIP-qPCR measurement of promoter-binding site occupancy over time using a two-parameter ODE model to better understand the regulatory causality and topology of the glucose responsive network in *H. salinarum.* We found that TrmB operates directly, indirectly and in conjunction with other factors to regulate gene expression levels and temporal dynamics in response to glucose.

The cobalamin biosynthesis pathway appears to be regulated transcriptionally both by TrmB and a TrmB-dependent secondary regulator. Using logic approximations, we show that FFL topology is consistent with our data (Figure 8B). Previous ChIP-chip data also suggest that TrmB binds the promoters of four TFs: VNG0156C, VNG0247C, VNG0878G and VNG1179C (17). The TrmB *cis*-regulatory motif has been identified in the upstream regions of several more TFs, including VNG1899G. To determine which of these TrmB-dependent TFs are the most likely candidates for the secondary regulator, we explored the Environmental and Gene Regulatory Influence Network (EGRIN). EGRIN is a computationally inferred GRN for *H. salinarum* learned from gene expression data across a range of genetic and environmental conditions (14). Of the directly TrmB regulated and potentially TrmB-regulated TFs, we predict from EGRIN that VNG0156C, VNG1899G or VNG1179C are more likely candidates for the regulation of cobalamin biosynthesis.

Alternatively, feed-forward topology could be achieved *via* feedback from a secondary metabolite, such as adenosylcobalamin (vitamin B-12), rather than direct regulation by another TF. For example, in *Salmonella typhimurium* B-12 biosynthetic enzymes are regulated by riboswitches. These 5′ untranslated region mRNA elements bind to B-12 with high affinity to control translation of target mRNAs (36).

It is also possible that a more complicated regulatory topology underlies the dynamics we observed. For instance, in *S.typhimurium*, transcriptional control of B-12 biosynthesis involves the interaction of several general and specific TFs. Genes coding for enzymes in B-12 synthesis appear to be transcriptionally activated by the relevant global TF, Crp (*via* cAMP), under aerobic conditions and ArcA/ArcB under anaerobic conditions. Besides affecting the transcription of the *cob*/*cbi* genes, the condition-appropriate global regulator also activates PocR. PocR binds DNA and further activates cobalamin synthesis and propanediol metabolism [the pathway for which cobalamin is needed; (37)].

Despite the potential for complicated control systems, we favor the transcriptional FFL network proposed for *cob/cbi* regulation. Riboswitch motifs have been identified in numerous bacteria, but few archaea (38). Computational analysis of the *H. salinarum* transcriptome reveals little evidence for extensive 5′ untranslated regions (39) or riboswitch motifs. Furthermore, the timing of the feed-forward dynamics is consistent with a transcriptional feed-forward system rather than with more rapid post-transcriptional regulation. Although it remains formally possible that other more complex regulatory topologies involving TrmB are acting on the B-12 biosynthesis cluster, our data most parsimoniously support the less complex FFL motif model under the conditions tested here.

The FFL motif is widely distributed in GRNs across the bacterial and eukaryotic domains. In *Escherichia coli,* FFL motifs are statistically overrepresented in network analyses (40). For example, the global TF Crp and the specific TF AraC control the arabinose utilization operon in a FFL motif with AND logic (40); *i.e.* to transcribe the arabinose utilization genes, the lack of glucose AND the presence of arabinose must be sensed. FFL motifs are similarly overrepresented in yeast (41). Although relatively few putative FFLs have been specifically identified in archaea (28), many of the same evolutionary drivers that have led to feed-forward motifs in other genomes may also be relevant (42).

Purine biosynthesis, on the other hand, appears to be co-regulated by a non-TrmB-dependent TF in *H. salinarum* (Figure 4). The EGRIN network suggests several other regulators may be in play, including VNG5009H, VNG2614H and VNG2163H; however, none of these are directly TrmB regulated. TrmB is required for the appropriate equilibrium level of these genes, suggesting that it plays at least a partial role in their regulation (Supplementary Figure S2). In *E. coli* and *Bacillus subtilis,* for example, purine biosynthesis is transcriptionally regulated by the PurR repressor in response to the small molecule hypoxanthine (43,44). Hypoxanthine integrates exogenous purine availability, salvage and *de novo* biosynthesis to maintain cellular purine levels. Although we could not identify a PurR homologue in *H. salinarum* on the basis of sequence, an analogous topology seems likely based on our analysis.

By integrating dynamic measurements of gene expression with promoter occupancy, this study has provided insight into TrmB-mediated transcriptional regulation of metabolism. Previous studies on TrmB in archaea have focused on steady-state gene expression and promoter-binding site occupancy to determine network structure and infer regulatory relationships (17,45). Under steady-state analysis, it is difficult to discern the role of TFs in activating or repressing genes because the signal from primary TFs is frequently confused by secondary regulation. For example, it is difficult to infer the sign of edges in an FFL (activation or repression), as the equilibrium level of gene expression output is affected by both input edges. We have demonstrated that adding a temporal dimension to gene expression measurement during metabolic adjustment deconvolves such regulatory relationships. Using a network inference approach, we hypothesize a FFL regulatory topology from the signal at observable nodes. By capturing the temporal separation of the activity of primary and secondary regulators, we lay the groundwork for establishing regulatory causality. This method of dynamic measurement of gene expression in response to genetic and environmental perturbation may be a generally feasible method for reconstructing GRNs in other organisms across all three domains of life.

## ACCESSION NUMBERS

NanoString data can be accessed under GEO series GSE47201 and platform GPL17188.

## SUPPLEMENTARY DATA

Supplementary Data are available at NAR Online.

Supplementary Data
