# Supplementary material for: Protein–DNA binding dynamics predict transcriptional response to nutrients in archaea
Source: Nucleic Acids Res. 2013 Jul 26;41(18):8546–58. doi: 10.1093/nar/gkt659 (PMC3794607; doi:10.1093/nar/gkt659)
Supplement: Supplementary Data [file supp_41_18_8546__index.html]

Protein–DNA binding dynamics predict transcriptional response to nutrients in archaea — Supplementary Data 

# Protein–DNA binding dynamics predict transcriptional response to nutrients in archaea

## Supplementary Data

files

**Files in this Data Supplement:**

- Supplementary Data - pdf file
- Supplementary Data - pdf file
- Supplementary Data - docx file
- Supplementary Data - csv file
- Supplementary Data - xlsx file
- Supplementary Data - xlsx file
