# Supplementary material for: Protein–DNA binding dynamics predict transcriptional response to nutrients in archaea
Source: Nucleic Acids Res. 2013 Jul 26;41(18):8546–58. doi: 10.1093/nar/gkt659 (PMC3794607; doi:10.1093/nar/gkt659)
Supplement: Supplementary Data [file supp_gkt659_Supplementary_Figure_1.3nar.pdf]

Supplementary Figure 1

Supplementary Figure 1. ODE model fit to NanoString gene expression data in response to glucose *Δura3* (parent strain) and *Δura3ΔtrmB* mutant background for all 100 genes in the dataset. Black lines represent mRNA level in the *Δura3* strain. Red lines represent mRNA level in the *Δura3ΔtrmB* strain. The grey line and the dotted orange line show model fits for *Δura3* and *Δura3ΔtrmB* respectively. Error bars are standard error from the average of 2 biological replicates of the gene expression data. Title color matches the *Δura3* clusters in Figure 4.

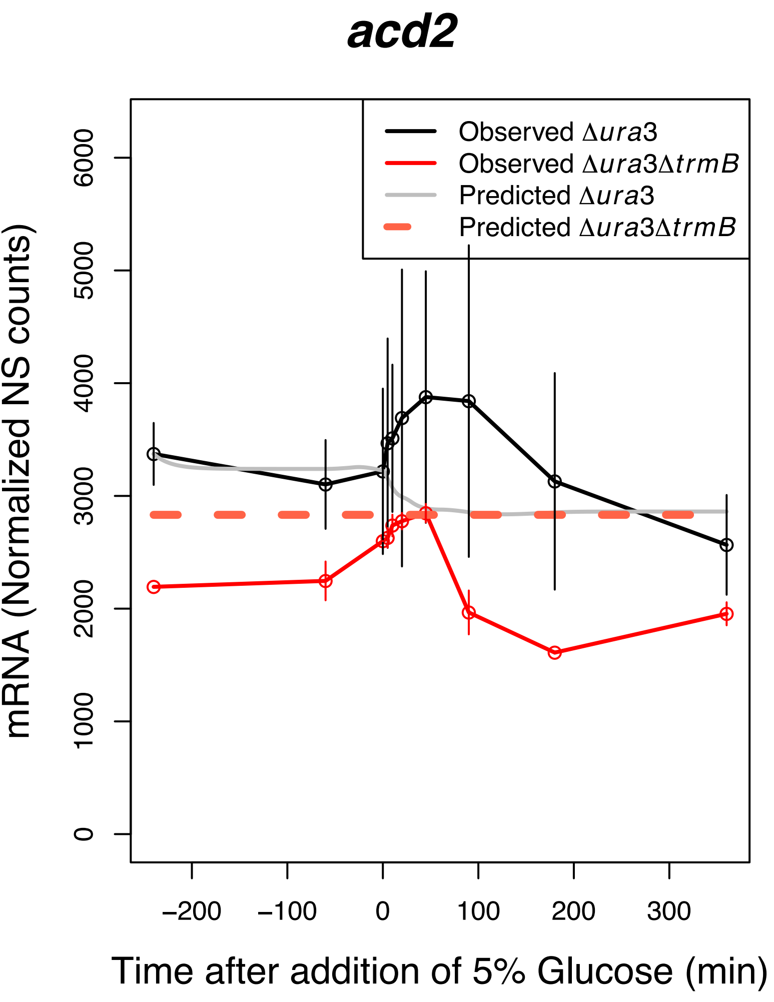

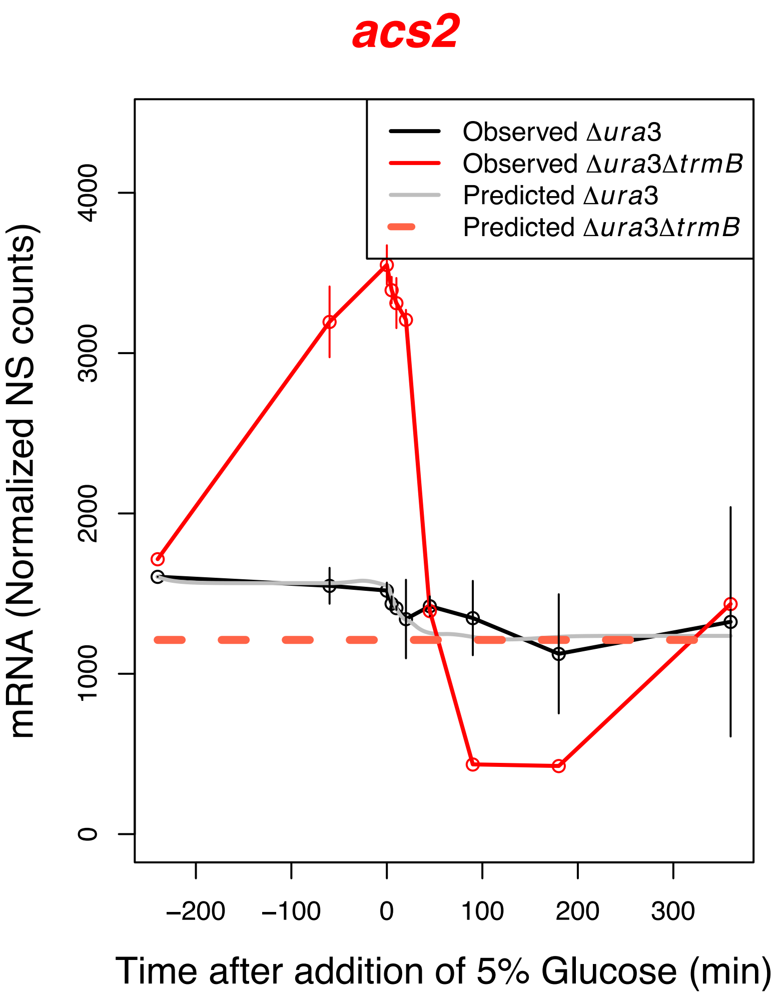

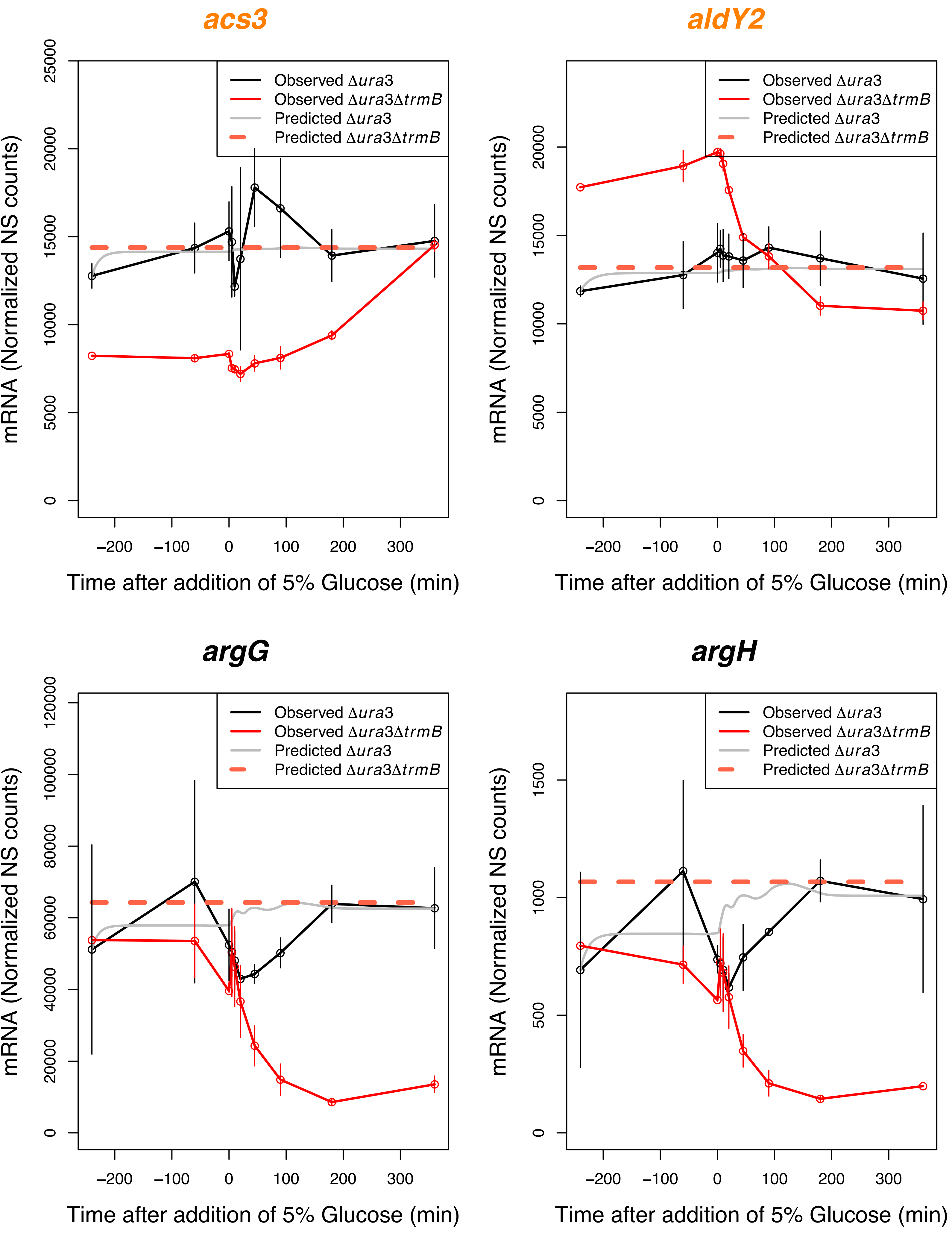
acs3
aldY2
argG
argH
Observed Δura3
Observed Δura3ΔtrmB
Predicted Δura3
Predicted Δura3ΔtrmB
mRNA (Normalized NS counts)
Time after addition of 5% Glucose (min)
−200
−100
0
100
200
300
0
5000
10000
15000
20000
25000
20000
40000
60000
80000
100000
120000
500
1000
1500

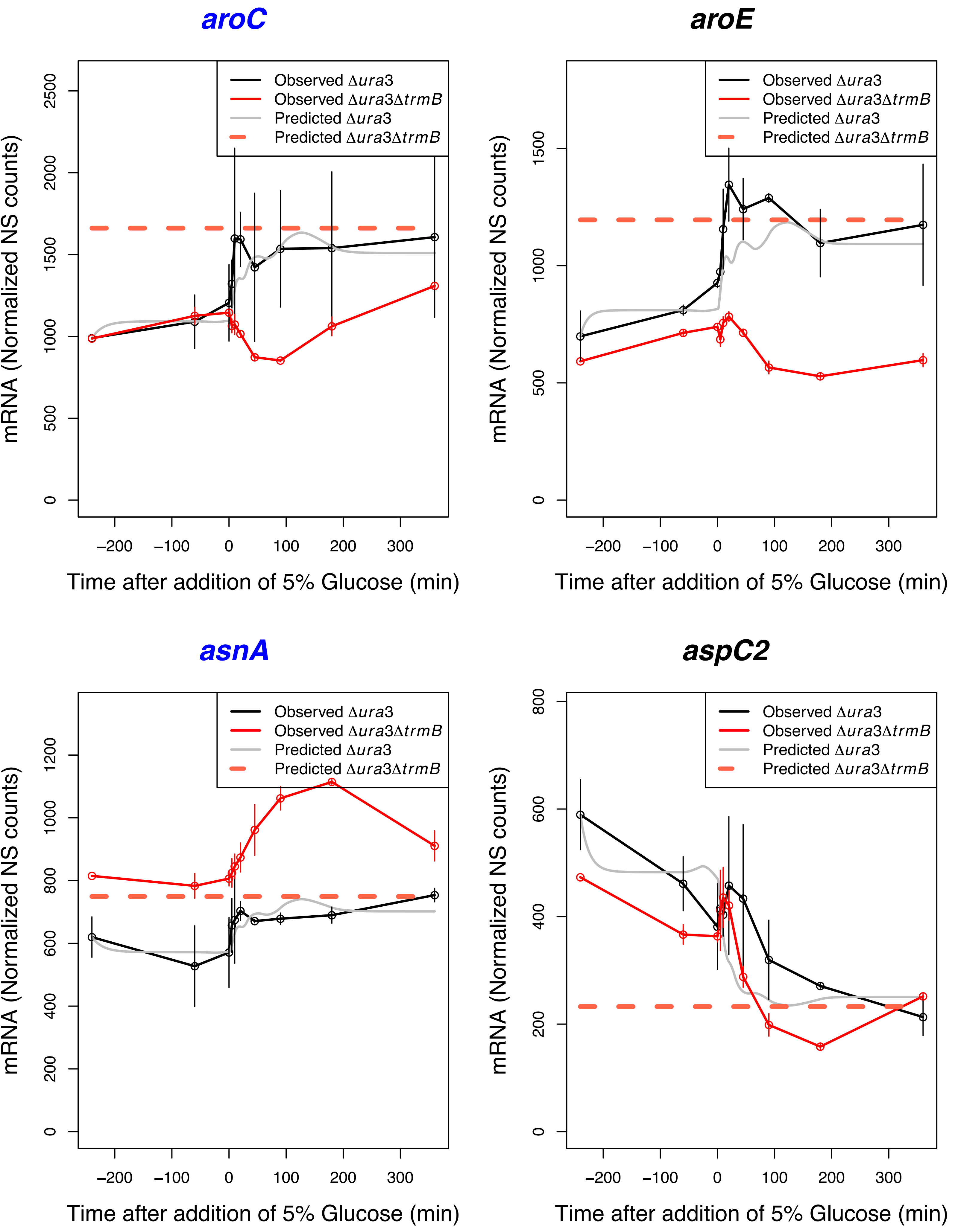

aroC
aroE
asnA
aspC2
Observed Δura3
Observed Δura3ΔtrmB
Predicted Δura3
Predicted Δura3ΔtrmB
mRNA (Normalized NS counts)
Time after addition of 5% Glucose (min)
0
500
1000
1500
2000
2500
200
400
600
800
1200
−200
−100
100
300

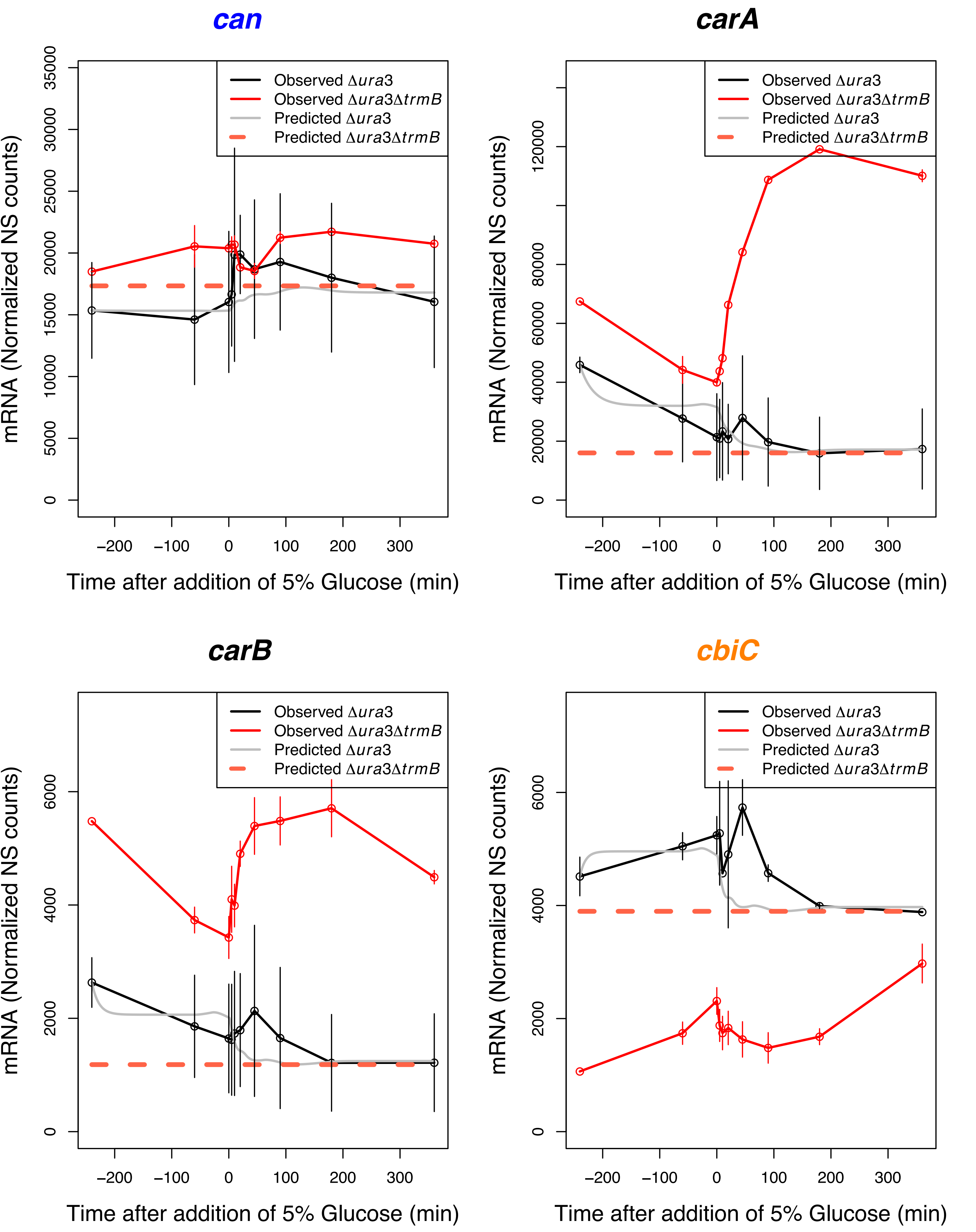
can
Observed Δura3
Observed Δura3ΔtrmB
Predicted Δura3
Predicted Δura3ΔtrmB
mRNA (Normalized NS counts)
0
5000
10000
15000
20000
25000
30000
35000
−200
−100
0
100
200
300
Time after addition of 5% Glucose (min)
carA
Observed Δura3
Observed Δura3ΔtrmB
Predicted Δura3
Predicted Δura3ΔtrmB
mRNA (Normalized NS counts)
0
20000
40000
60000
80000
120000
−200
−100
0
100
200
300
Time after addition of 5% Glucose (min)
carB
Observed Δura3
Observed Δura3ΔtrmB
Predicted Δura3
Predicted Δura3ΔtrmB
mRNA (Normalized NS counts)
0
2000
4000
6000
−200
−100
0
100
200
300
Time after addition of 5% Glucose (min)
cbiC
Observed Δura3
Observed Δura3ΔtrmB
Predicted Δura3
Predicted Δura3ΔtrmB
mRNA (Normalized NS counts)
0
2000
4000
6000
−200
−100
0
100
200
300
Time after addition of 5% Glucose (min)

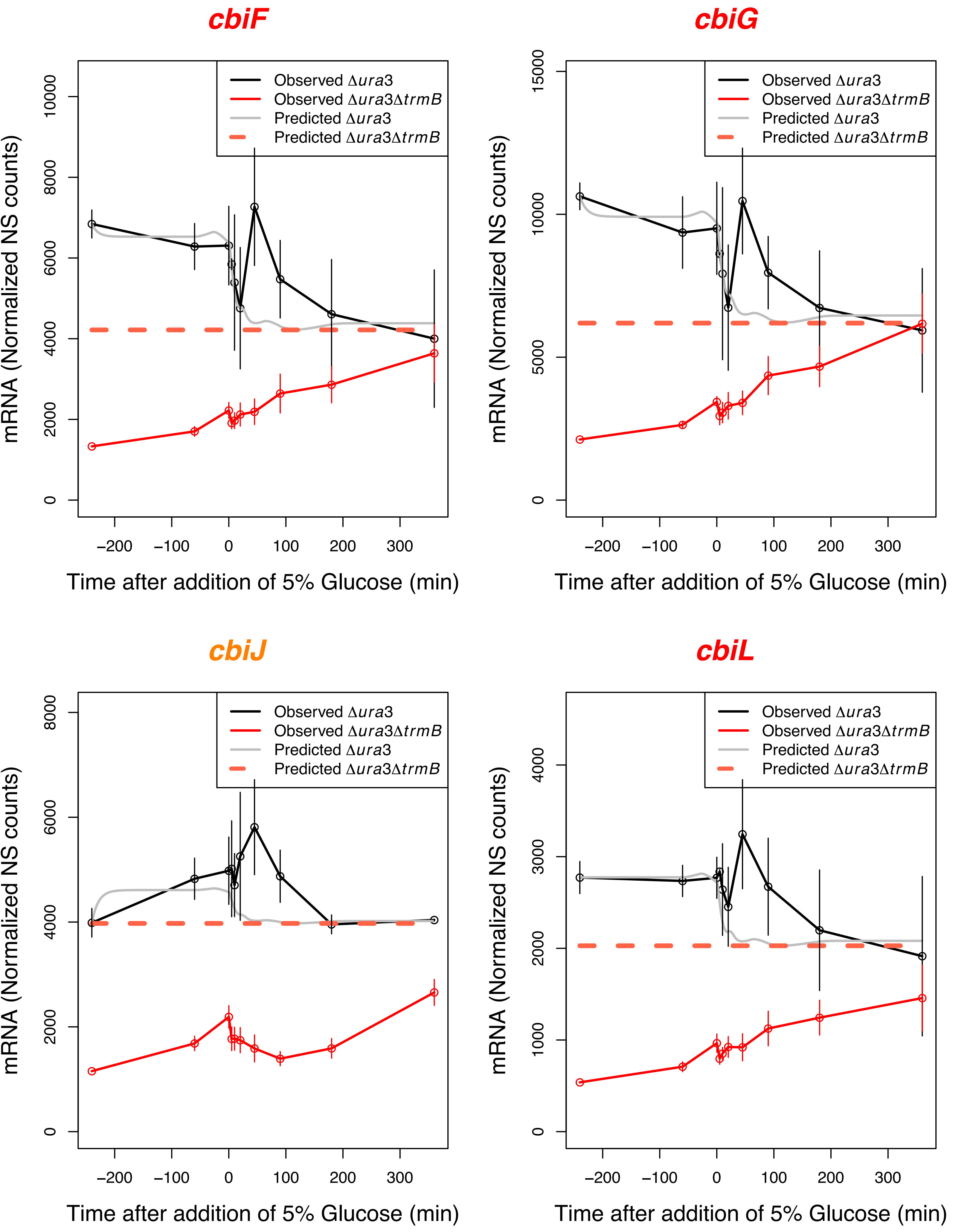

cbiF
Observed Δura3
Observed Δura3ΔtrmB
Predicted Δura3
Predicted Δura3ΔtrmB
mRNA (Normalized NS counts)
0
2000
4000
6000
8000
10000
−200
−100
0
100
200
300
Time after addition of 5% Glucose (min)
cbiG
Observed Δura3
Observed Δura3ΔtrmB
Predicted Δura3
Predicted Δura3ΔtrmB
mRNA (Normalized NS counts)
0
5000
10000
15000
−200
−100
0
100
200
300
Time after addition of 5% Glucose (min)
cbiJ
Observed Δura3
Observed Δura3ΔtrmB
Predicted Δura3
Predicted Δura3ΔtrmB
mRNA (Normalized NS counts)
0
2000
4000
6000
8000
−200
−100
0
100
200
300
Time after addition of 5% Glucose (min)
cbiL
Observed Δura3
Observed Δura3ΔtrmB
Predicted Δura3
Predicted Δura3ΔtrmB
mRNA (Normalized NS counts)
0
1000
2000
3000
4000
−200
−100
0
100
200
300
Time after addition of 5% Glucose (min)

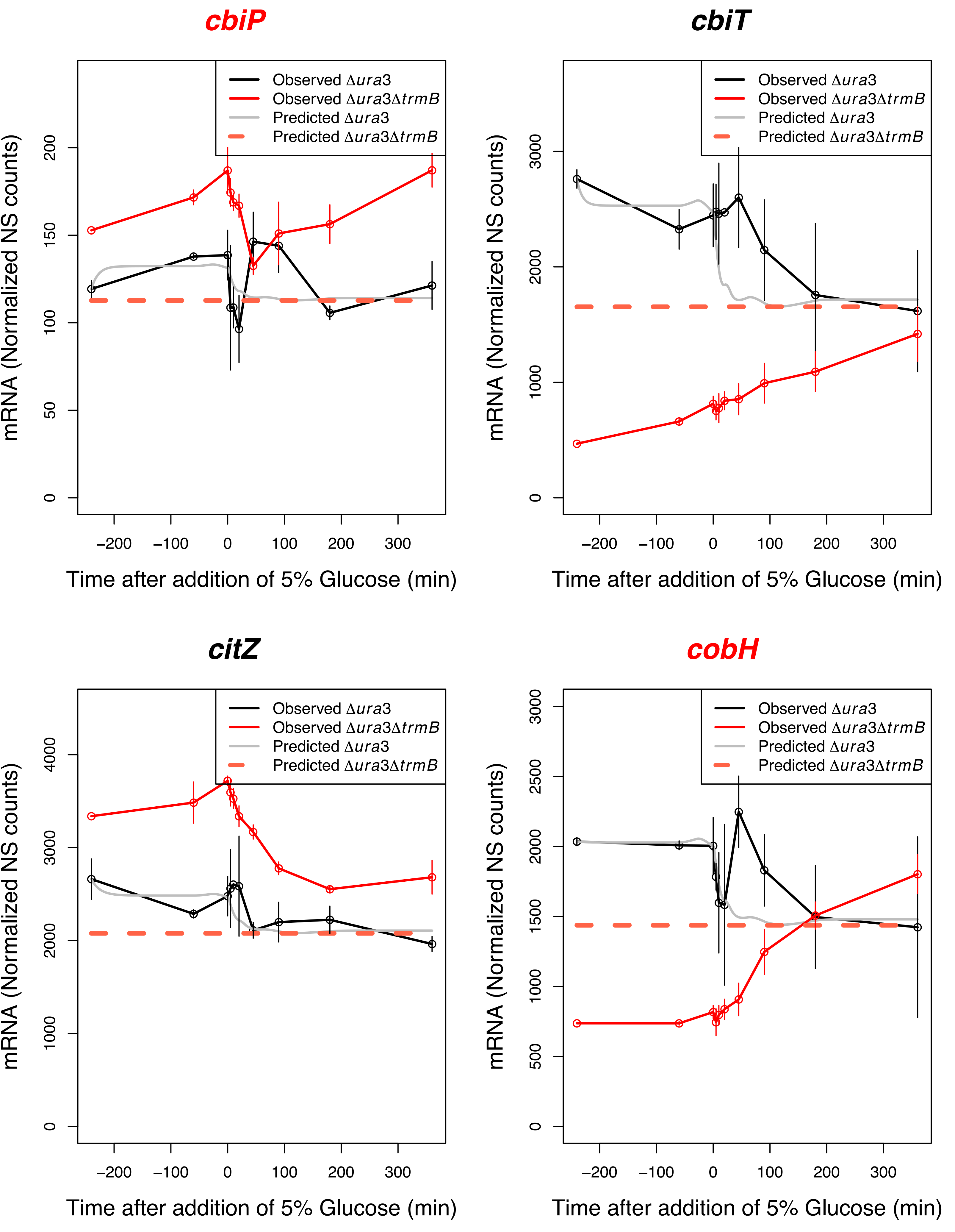

cbiP
cbiT
citZ
cobH
Observed Δura3
Observed Δura3ΔtrmB
Predicted Δura3
Predicted Δura3ΔtrmB
mRNA (Normalized NS counts)
Time after addition of 5% Glucose (min)

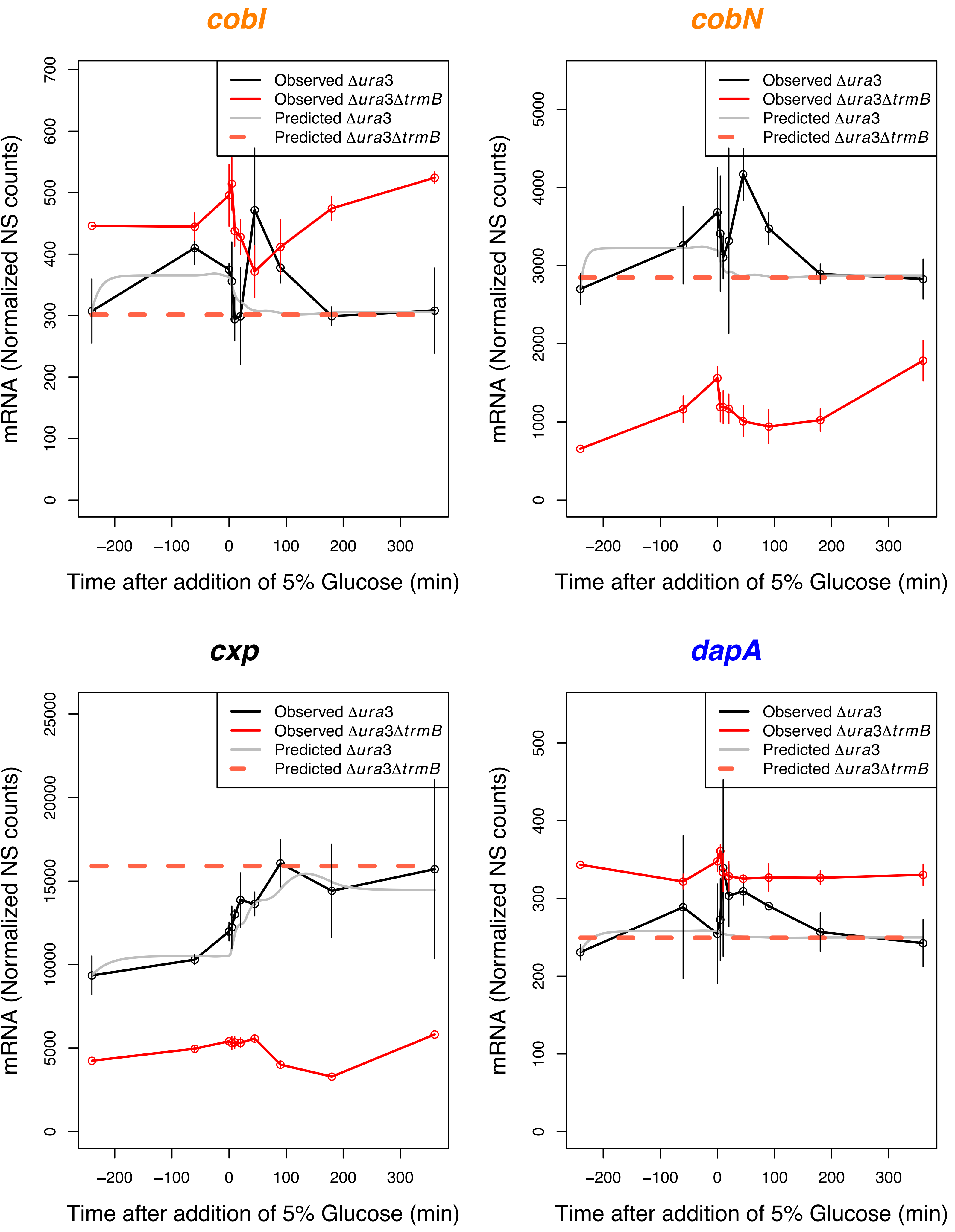
cobI
Observed Δura3
Observed Δura3ΔtrmB
Predicted Δura3
Predicted Δura3ΔtrmB
mRNA (Normalized NS counts)
Time after addition of 5% Glucose (min)
cobN
Observed Δura3
Observed Δura3ΔtrmB
Predicted Δura3
Predicted Δura3ΔtrmB
mRNA (Normalized NS counts)
Time after addition of 5% Glucose (min)
cxp
Observed Δura3
Observed Δura3ΔtrmB
Predicted Δura3
Predicted Δura3ΔtrmB
mRNA (Normalized NS counts)
Time after addition of 5% Glucose (min)
dapA
Observed Δura3
Observed Δura3ΔtrmB
Predicted Δura3
Predicted Δura3ΔtrmB
mRNA (Normalized NS counts)
Time after addition of 5% Glucose (min)

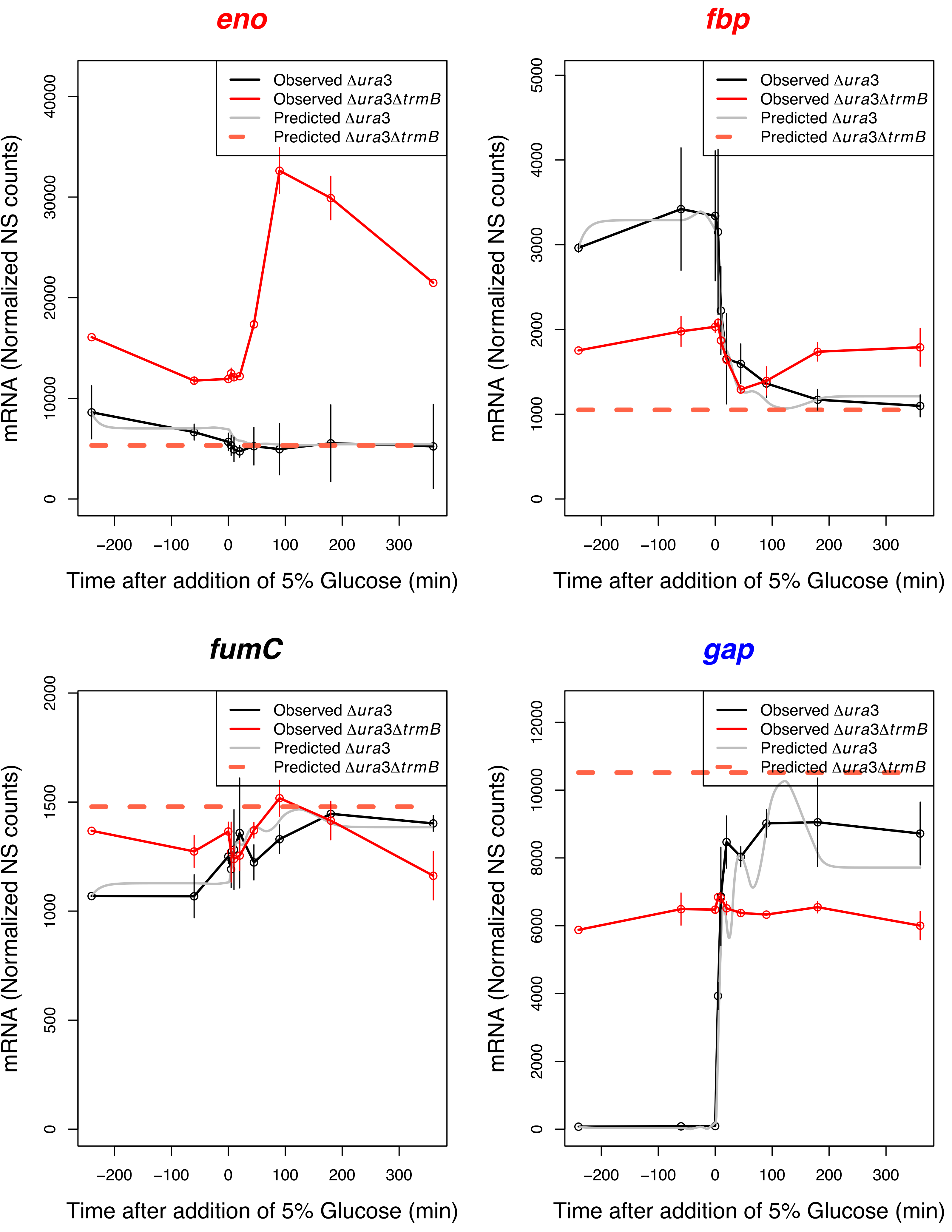

eno
Observed Δura3
Observed Δura3ΔtrmB
Predicted Δura3
Predicted Δura3ΔtrmB
mRNA (Normalized NS counts)
0
10000
20000
30000
40000
−200
−100
0
100
200
300
Time after addition of 5% Glucose (min)
fbp
Observed Δura3
Observed Δura3ΔtrmB
Predicted Δura3
Predicted Δura3ΔtrmB
mRNA (Normalized NS counts)
0
1000
2000
3000
4000
5000
−200
−100
0
100
200
300
Time after addition of 5% Glucose (min)
fumC
Observed Δura3
Observed Δura3ΔtrmB
Predicted Δura3
Predicted Δura3ΔtrmB
mRNA (Normalized NS counts)
0
500
1000
1500
2000
−200
−100
0
100
200
300
Time after addition of 5% Glucose (min)
gap
Observed Δura3
Observed Δura3ΔtrmB
Predicted Δura3
Predicted Δura3ΔtrmB
mRNA (Normalized NS counts)
0
2000
4000
6000
8000
10000
12000
−200
−100
0
100
200
300
Time after addition of 5% Glucose (min)

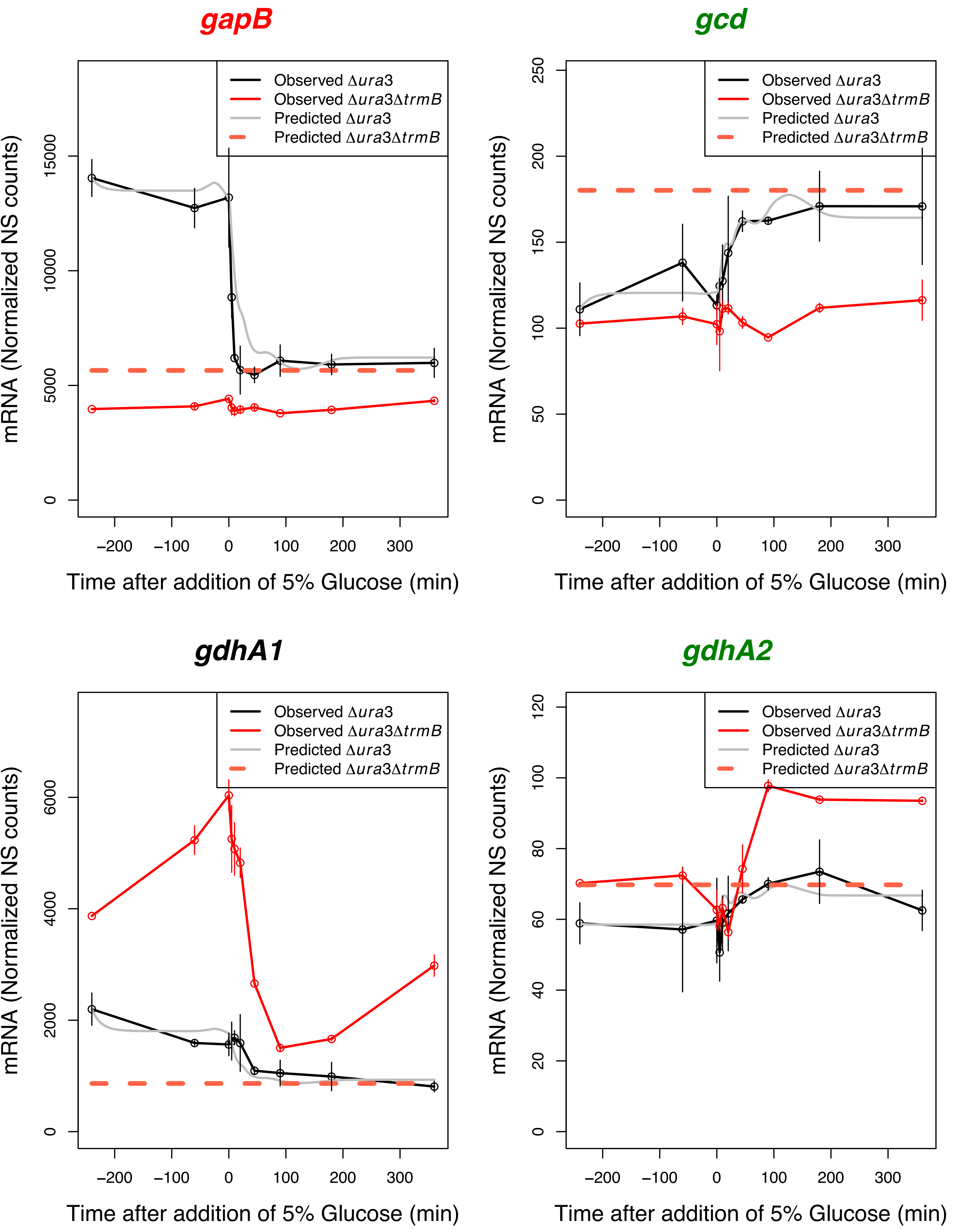
gapB
gcd
gdhA1
gdhA2
Observed Δura3
Observed Δura3ΔtrmB
Predicted Δura3
Predicted Δura3ΔtrmB
mRNA (Normalized NS counts)
Time after addition of 5% Glucose (min)
−200
−100
0
100
200
300
0
5000
10000
15000
50
150
200
250
2000
4000
6000
20
40
60
80
120

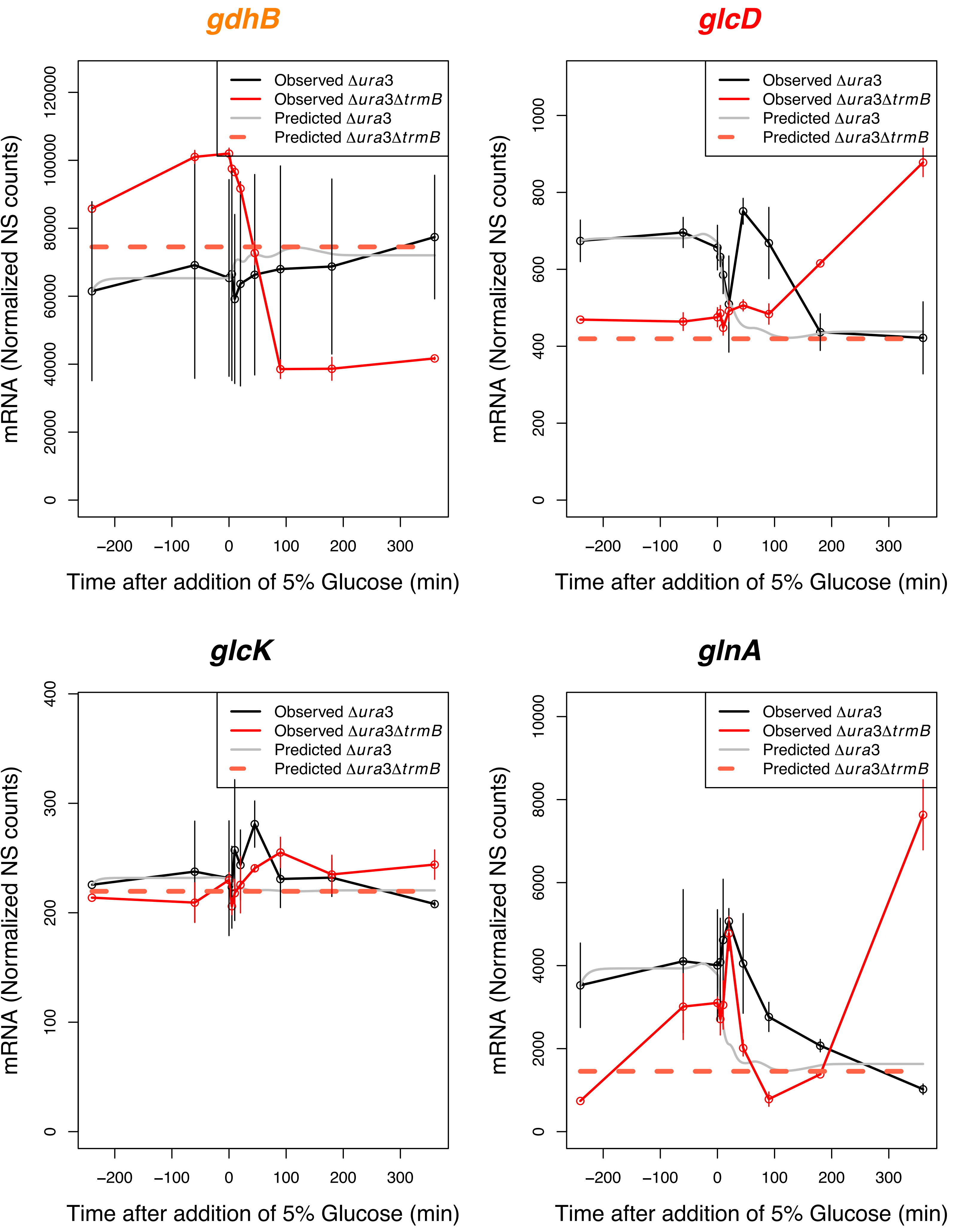
gdhB
Observed Δura3
Observed Δura3ΔtrmB
Predicted Δura3
Predicted Δura3ΔtrmB
mRNA (Normalized NS counts)
0
20000
40000
60000
80000
100000
120000
−200
−100
0
100
200
300
Time after addition of 5% Glucose (min)
glcD
Observed Δura3
Observed Δura3ΔtrmB
Predicted Δura3
Predicted Δura3ΔtrmB
mRNA (Normalized NS counts)
0
200
400
600
800
1000
−200
−100
0
100
200
300
Time after addition of 5% Glucose (min)
glcK
Observed Δura3
Observed Δura3ΔtrmB
Predicted Δura3
Predicted Δura3ΔtrmB
mRNA (Normalized NS counts)
0
100
200
300
400
−200
−100
0
100
200
300
Time after addition of 5% Glucose (min)
glnA
Observed Δura3
Observed Δura3ΔtrmB
Predicted Δura3
Predicted Δura3ΔtrmB
mRNA (Normalized NS counts)
0
2000
4000
6000
8000
10000
−200
−100
0
100
200
300
Time after addition of 5% Glucose (min)

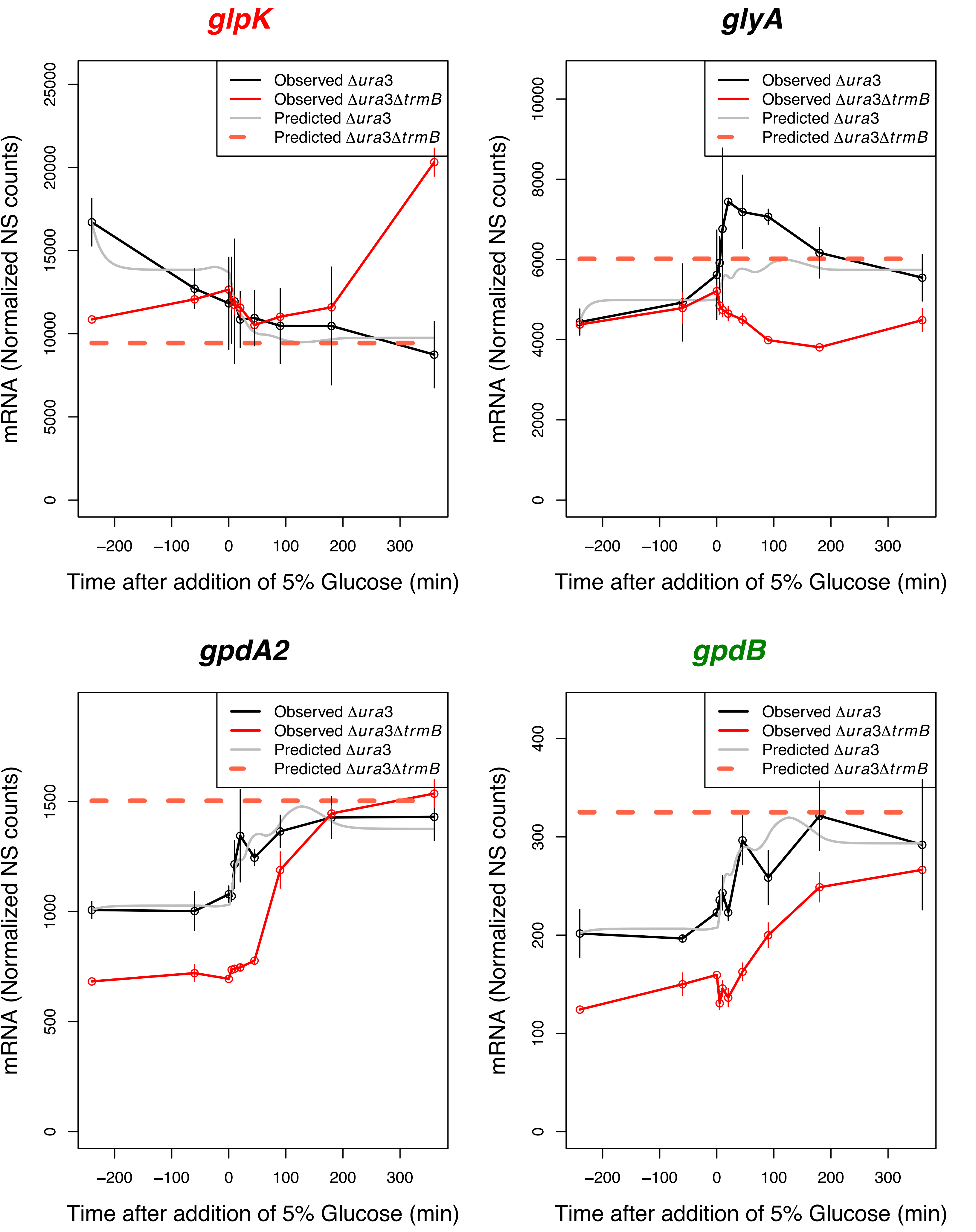

glpK
glyA
gpdA2
gpdB
Observed Δura3
Observed Δura3ΔtrmB
Predicted Δura3
Predicted Δura3ΔtrmB
mRNA (Normalized NS counts)
Time after addition of 5% Glucose (min)

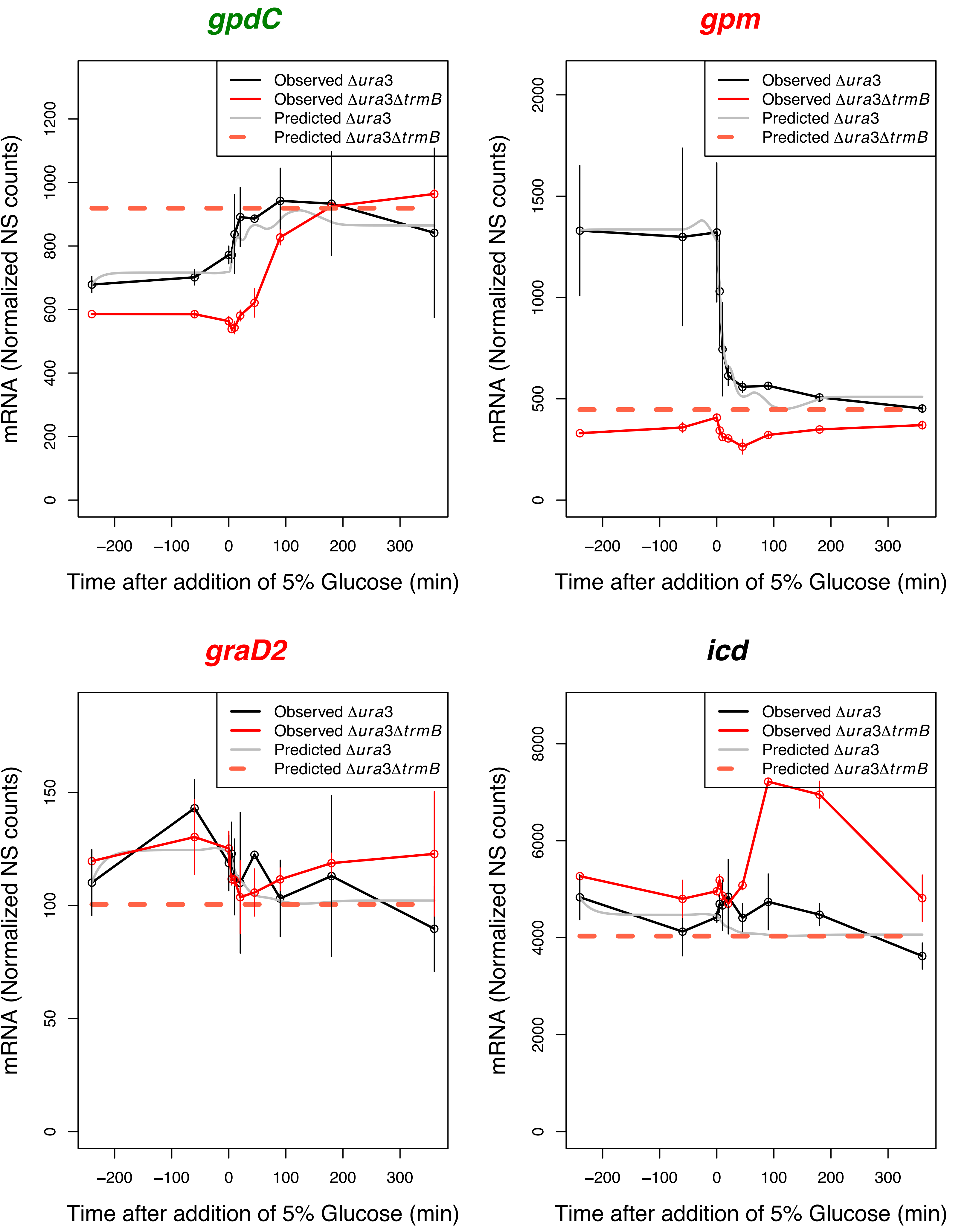
gpdC
gpm
graD2
icd
Observed Δura3
Observed Δura3ΔtrmB
Predicted Δura3
Predicted Δura3ΔtrmB
mRNA (Normalized NS counts)
Time after addition of 5% Glucose (min)

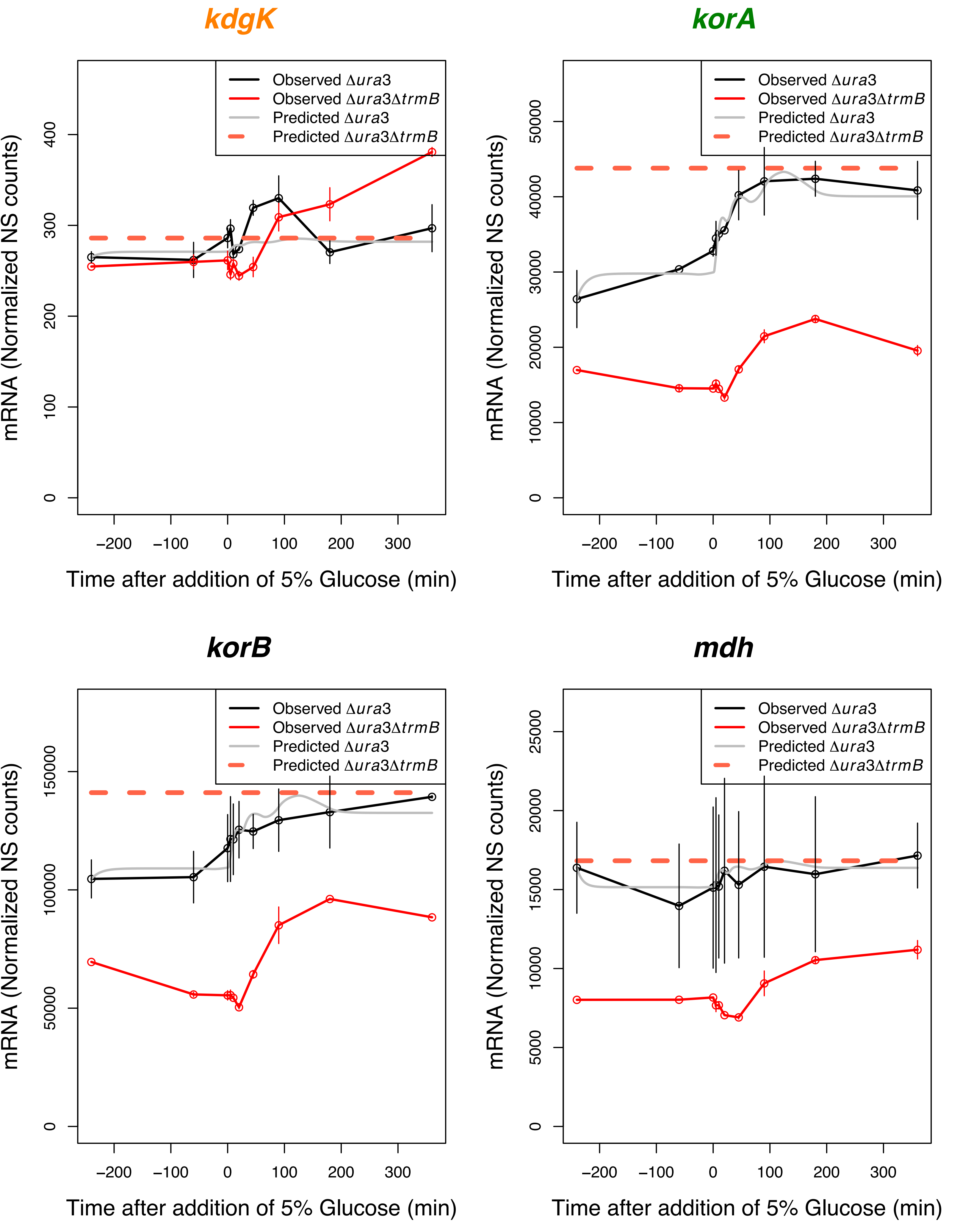
kdgK
korA
korB
mdh
Observed Δura3
Observed Δura3ΔtrmB
Predicted Δura3
Predicted Δura3ΔtrmB
mRNA (Normalized NS counts)
Time after addition of 5% Glucose (min)
−200
−100
0
100
200
300
400
10000
20000
30000
40000
50000
50000
100000
150000
5000
15000
25000

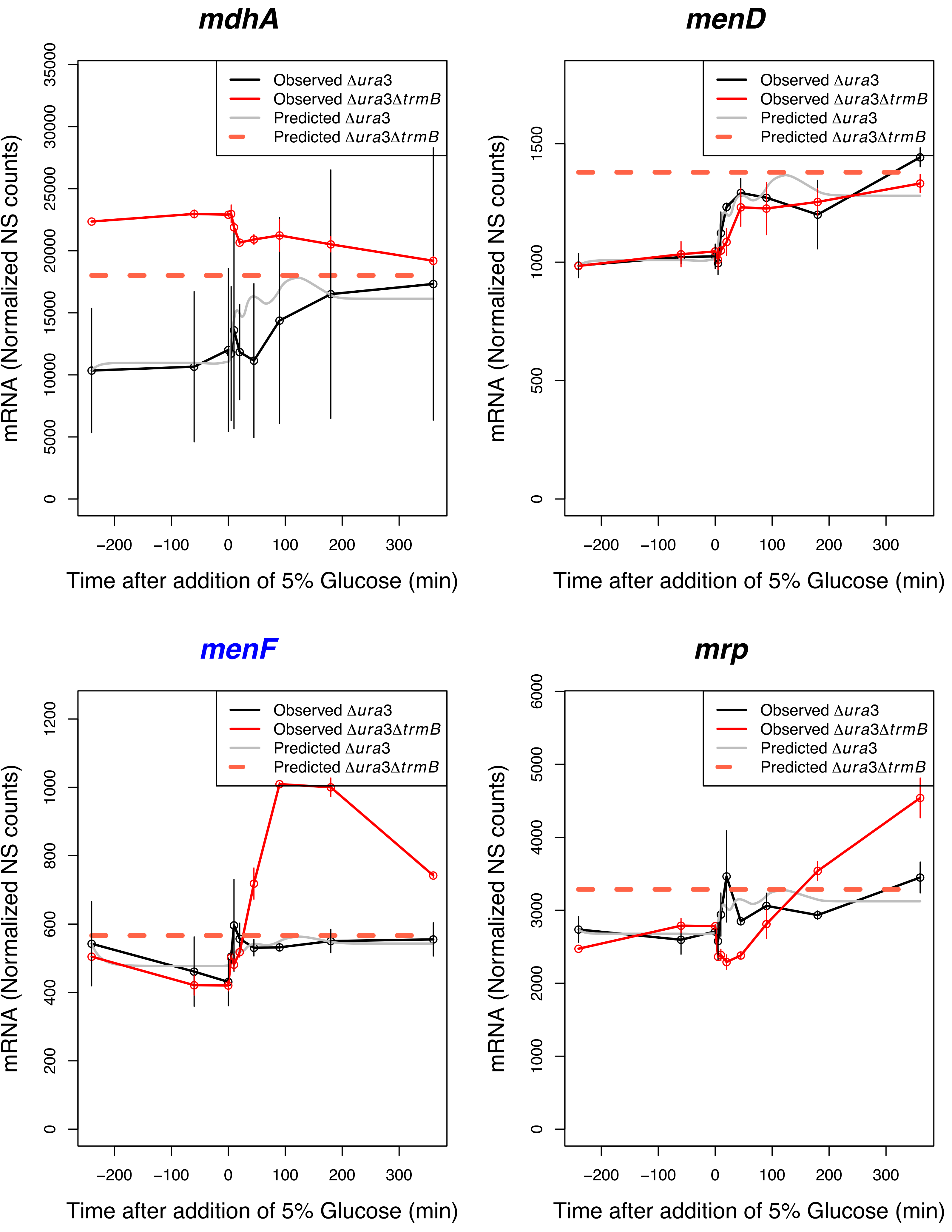
mdhA
menD
menF
mrp
Observed Δura3
Observed Δura3ΔtrmB
Predicted Δura3
Predicted Δura3ΔtrmB
mRNA (Normalized NS counts)
Time after addition of 5% Glucose (min)
−200
−100
0
100
200
300
0
5000
10000
15000
20000
25000
30000
35000
500
1000
1500
200
400
600
800
1200
2000
3000
4000
5000
6000

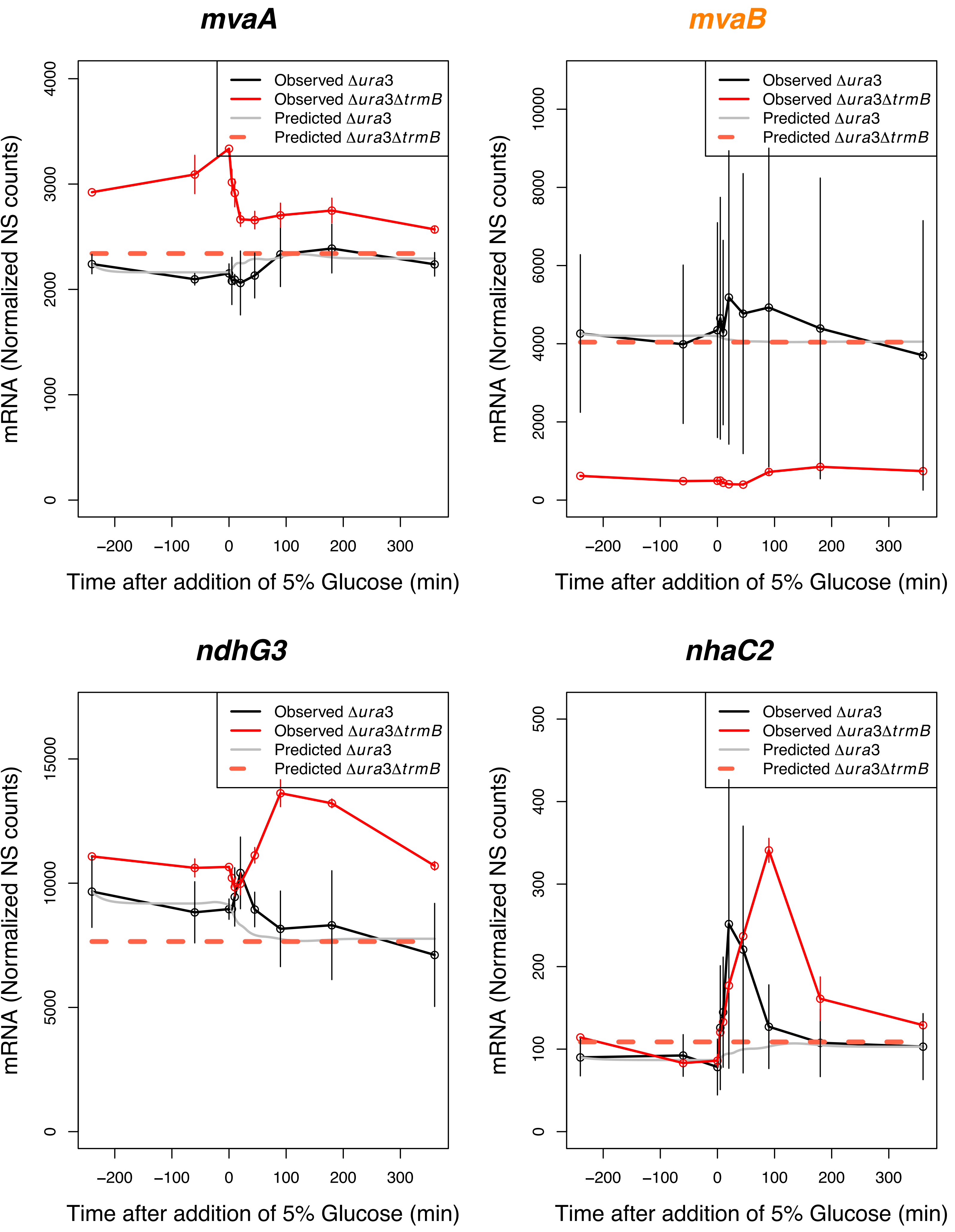
mvaA
Observed Δura3
Observed Δura3ΔtrmB
Predicted Δura3
Predicted Δura3ΔtrmB
mRNA (Normalized NS counts)
0
1000
2000
3000
4000
−200
−100
0
100
200
300
Time after addition of 5% Glucose (min)
mvaB
Observed Δura3
Observed Δura3ΔtrmB
Predicted Δura3
Predicted Δura3ΔtrmB
mRNA (Normalized NS counts)
0
2000
4000
6000
8000
10000
−200
−100
0
100
200
300
Time after addition of 5% Glucose (min)
ndhG3
Observed Δura3
Observed Δura3ΔtrmB
Predicted Δura3
Predicted Δura3ΔtrmB
mRNA (Normalized NS counts)
0
5000
10000
15000
−200
−100
0
100
200
300
Time after addition of 5% Glucose (min)
nhaC2
Observed Δura3
Observed Δura3ΔtrmB
Predicted Δura3
Predicted Δura3ΔtrmB
mRNA (Normalized NS counts)
0
100
200
300
400
500
−200
−100
0
100
200
300
Time after addition of 5% Glucose (min)

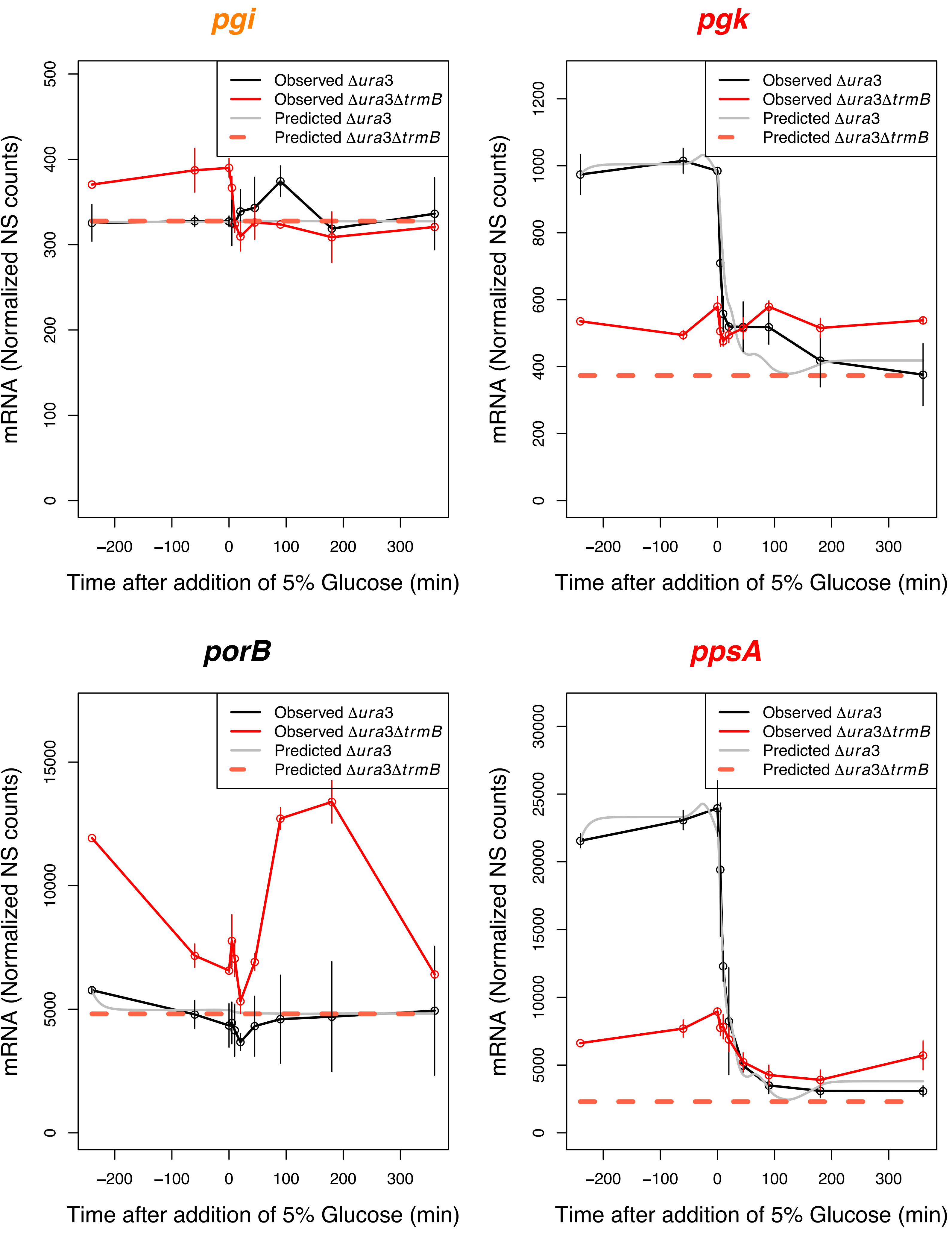
pgi
pgk
porB
ppsA
Observed Δura3
Observed Δura3ΔtrmB
Predicted Δura3
Predicted Δura3ΔtrmB
mRNA (Normalized NS counts)
Time after addition of 5% Glucose (min)
−200
−100
0
100
200
300
400
500
600
800
1000
1200
5000
10000
15000
20000
25000
30000

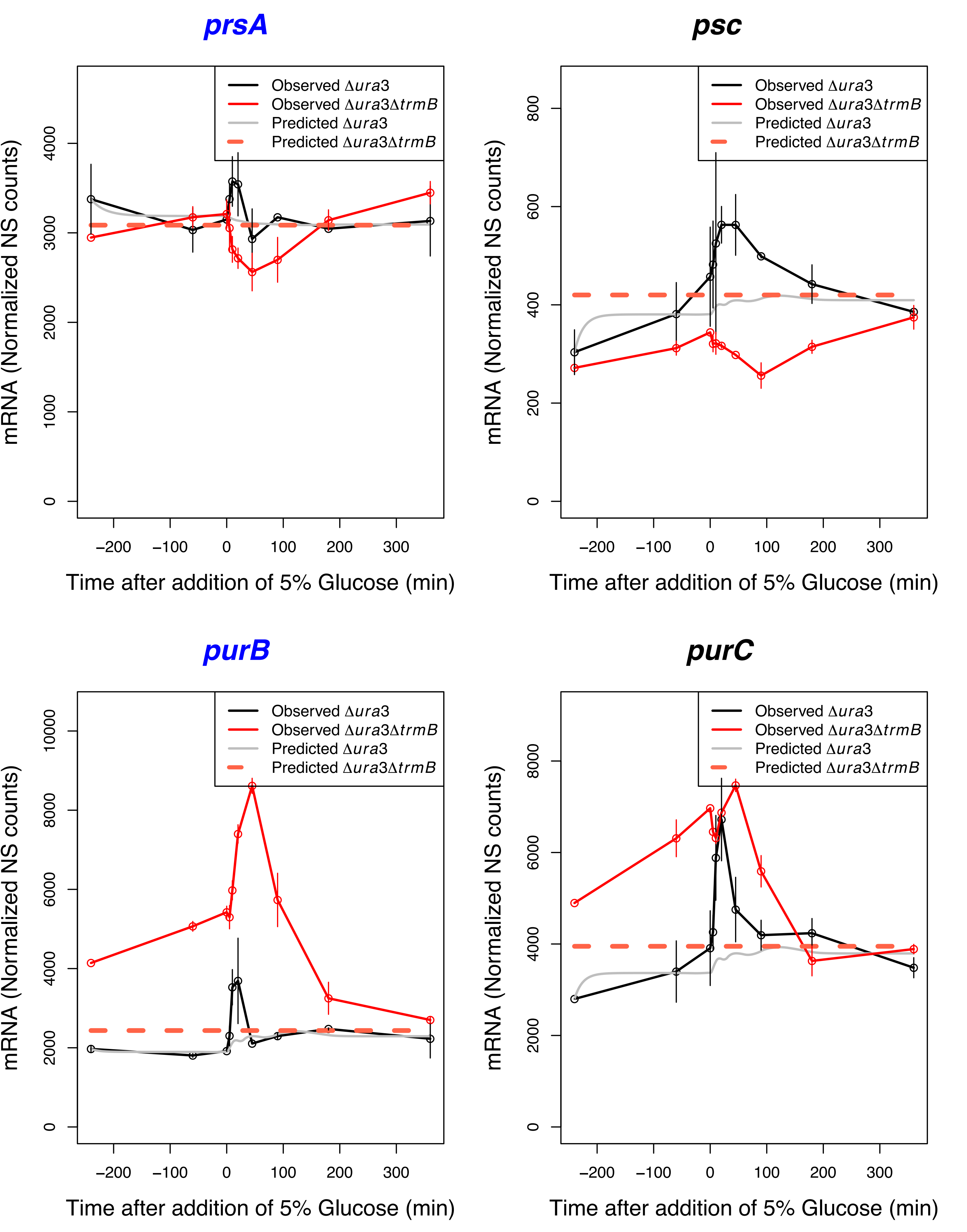

prsA
psc
purB
purC
Observed Δura3
Observed Δura3ΔtrmB
Predicted Δura3
Predicted Δura3ΔtrmB
mRNA (Normalized NS counts)
Time after addition of 5% Glucose (min)
−200
−100
0
100
200
300
0
1000
2000
3000
4000
200
400
600
800
6000
8000
10000

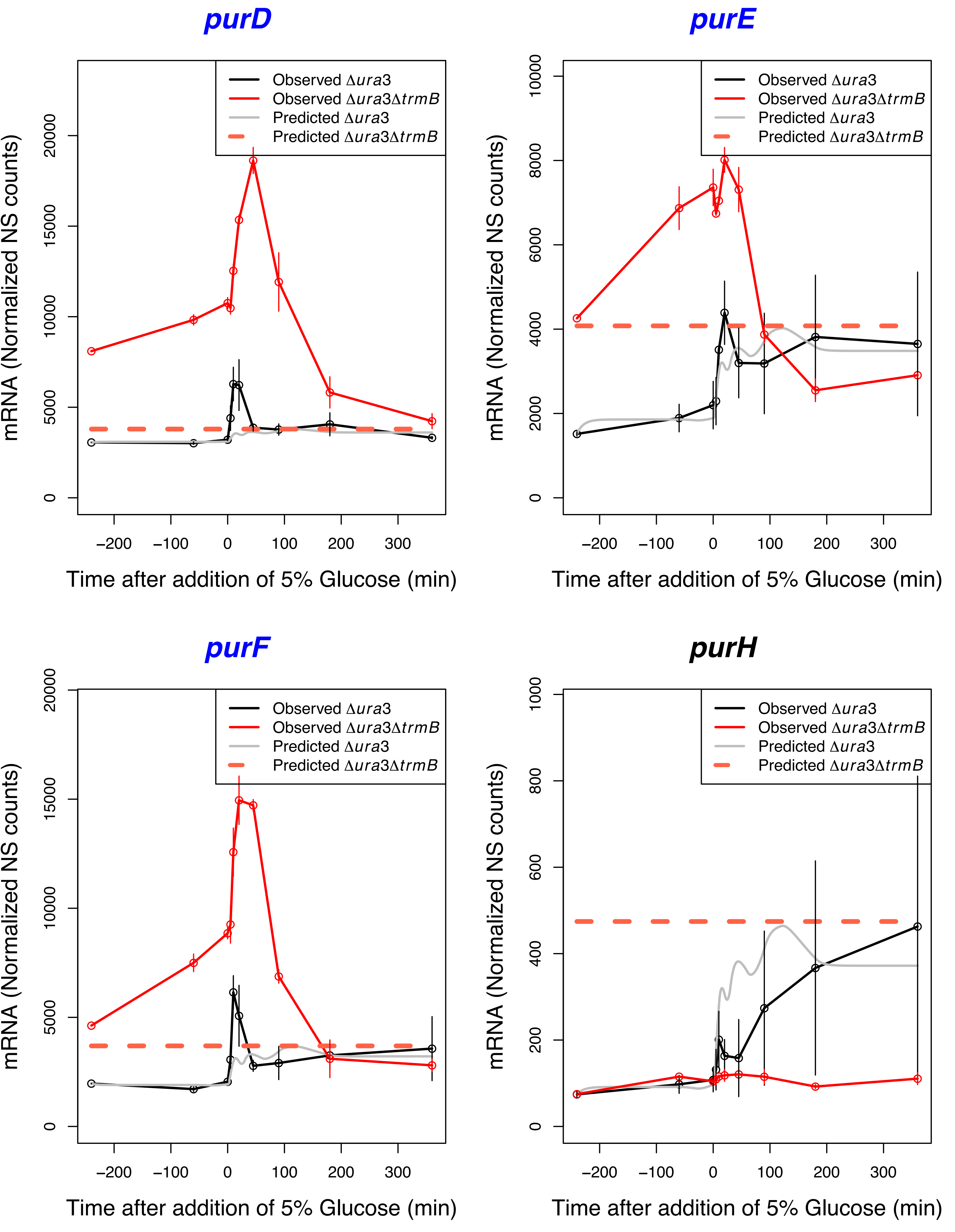

purD
purE
purF
purH
Observed Δura3
Observed Δura3ΔtrmB
Predicted Δura3
Predicted Δura3ΔtrmB
mRNA (Normalized NS counts)
Time after addition of 5% Glucose (min)
−200
−100
0
100
200
300
0
5000
10000
15000
20000
2000
4000
6000
8000
10000
200
400
600
800
1000

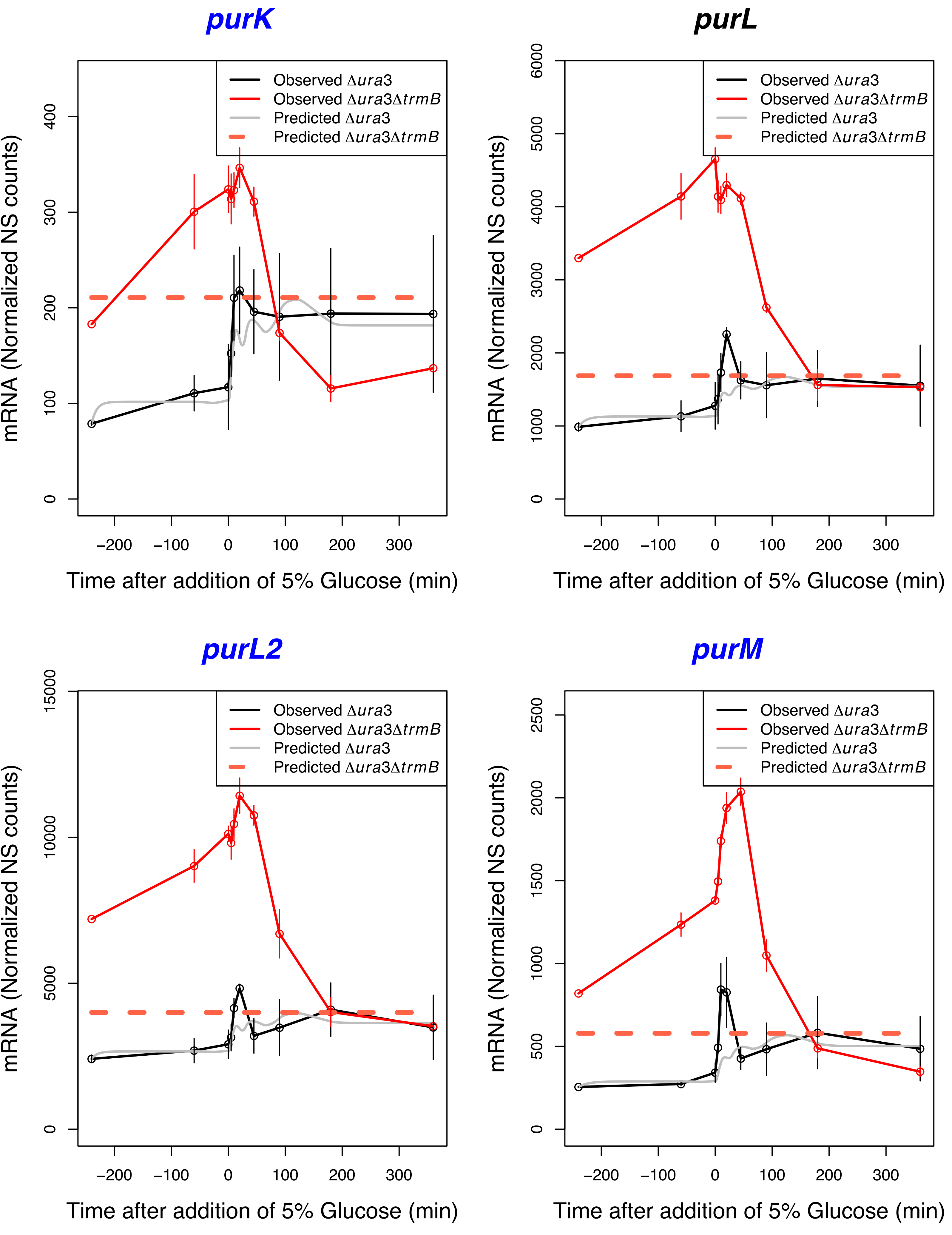
purK
purL
purL2
purM
Observed Δura3
Observed Δura3ΔtrmB
Predicted Δura3
Predicted Δura3ΔtrmB
mRNA (Normalized NS counts)
Time after addition of 5% Glucose (min)
−200
−100
0
100
200
300
400
1000
2000
3000
4000
5000
6000
5000
10000
15000
500
1000
1500
2000
2500

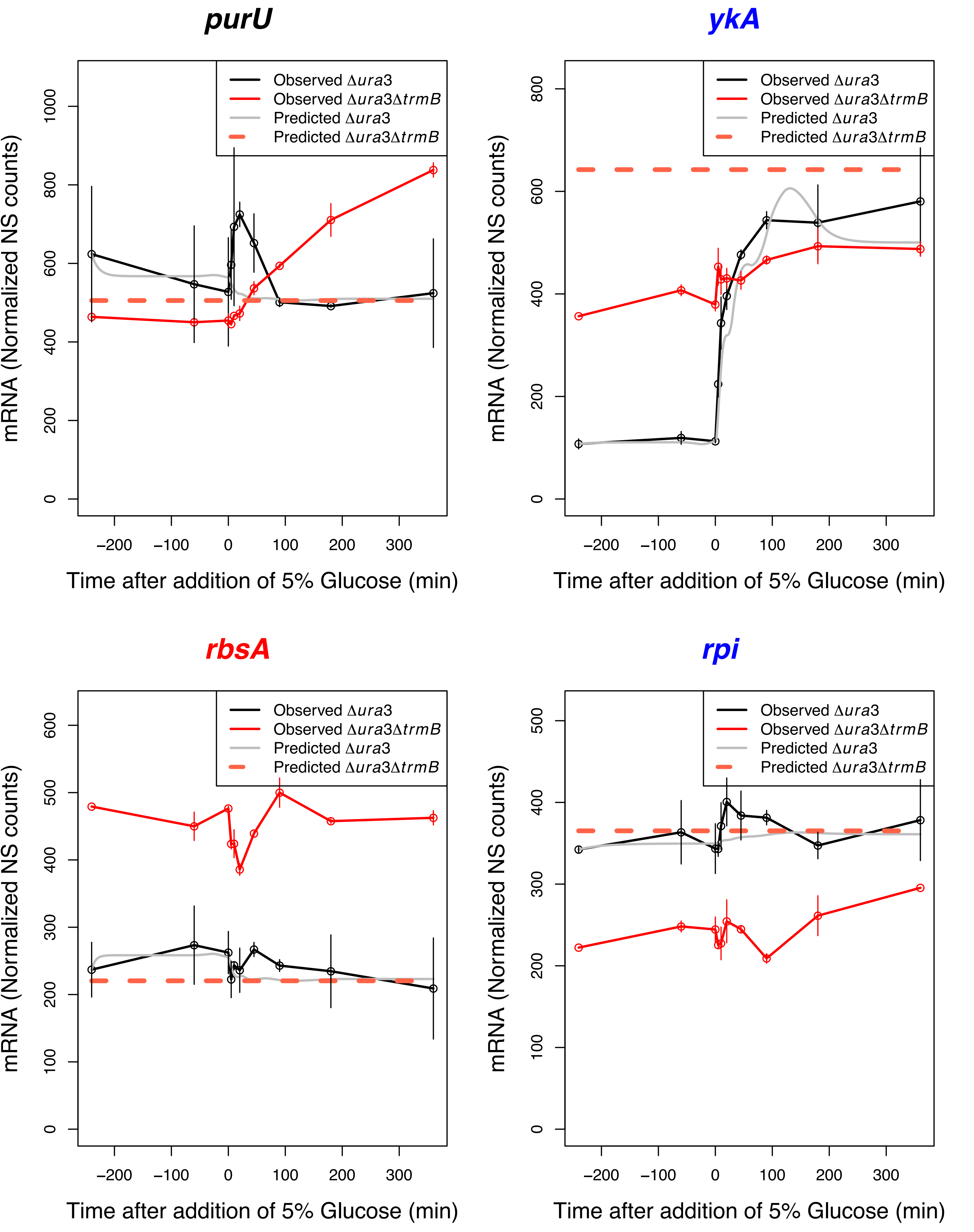
purU
ykA
rbsA
rpi
Observed Δura3
Observed Δura3ΔtrmB
Predicted Δura3
Predicted Δura3ΔtrmB
mRNA (Normalized NS counts)
Time after addition of 5% Glucose (min)
−200
−100
0
100
200
300
0
200
400
600
800
1000
500

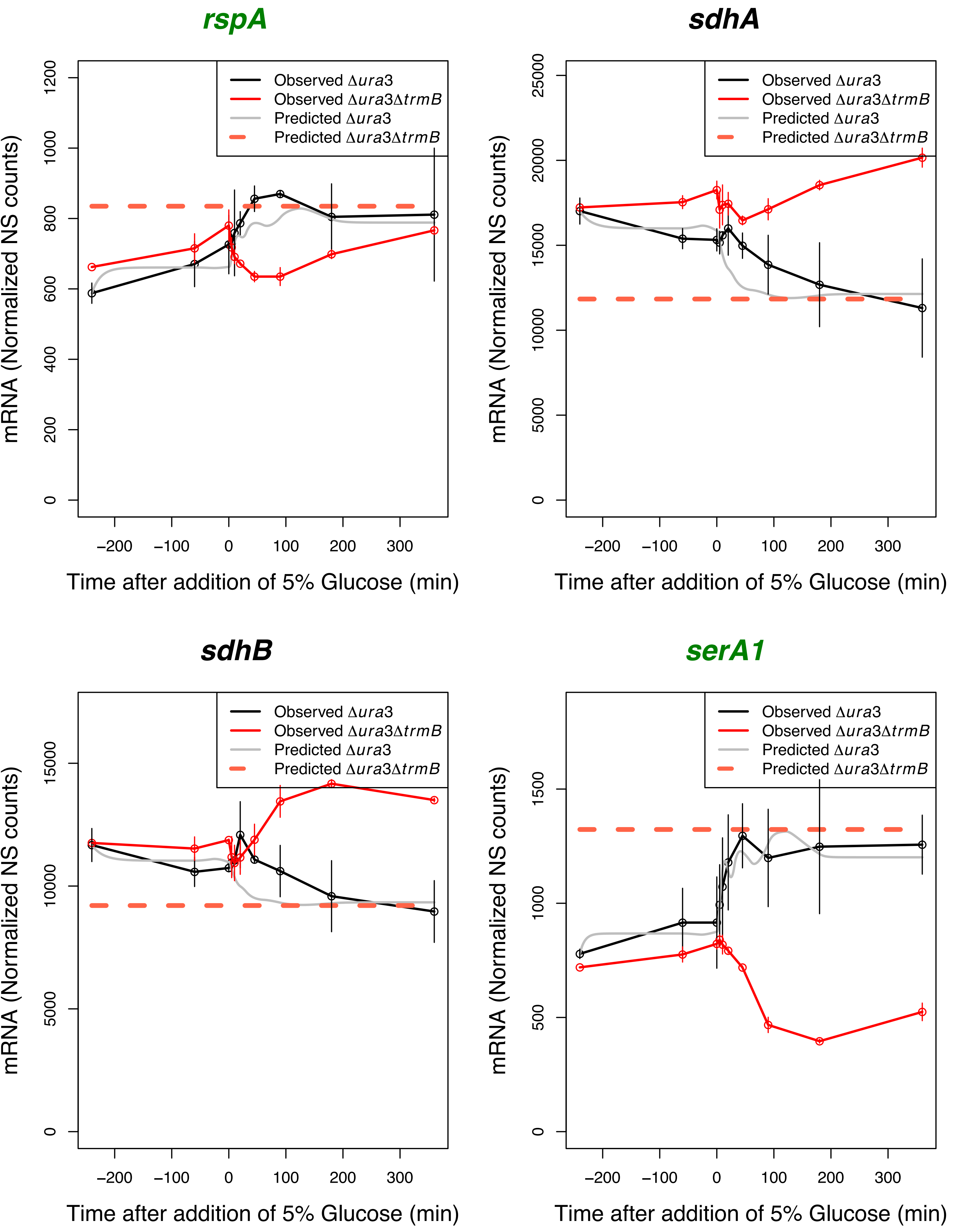
rspA
Observed Δura3
Observed Δura3ΔtrmB
Predicted Δura3
Predicted Δura3ΔtrmB
mRNA (Normalized NS counts)
0
200
400
600
800
1000
1200
−200
−100
0
100
200
300
Time after addition of 5% Glucose (min)
sdhA
Observed Δura3
Observed Δura3ΔtrmB
Predicted Δura3
Predicted Δura3ΔtrmB
mRNA (Normalized NS counts)
0
5000
10000
15000
20000
25000
−200
−100
0
100
200
300
Time after addition of 5% Glucose (min)
sdhB
Observed Δura3
Observed Δura3ΔtrmB
Predicted Δura3
Predicted Δura3ΔtrmB
mRNA (Normalized NS counts)
0
5000
10000
15000
−200
−100
0
100
200
300
Time after addition of 5% Glucose (min)
serA1
Observed Δura3
Observed Δura3ΔtrmB
Predicted Δura3
Predicted Δura3ΔtrmB
mRNA (Normalized NS counts)
0
500
1000
1500
−200
−100
0
100
200
300
Time after addition of 5% Glucose (min)

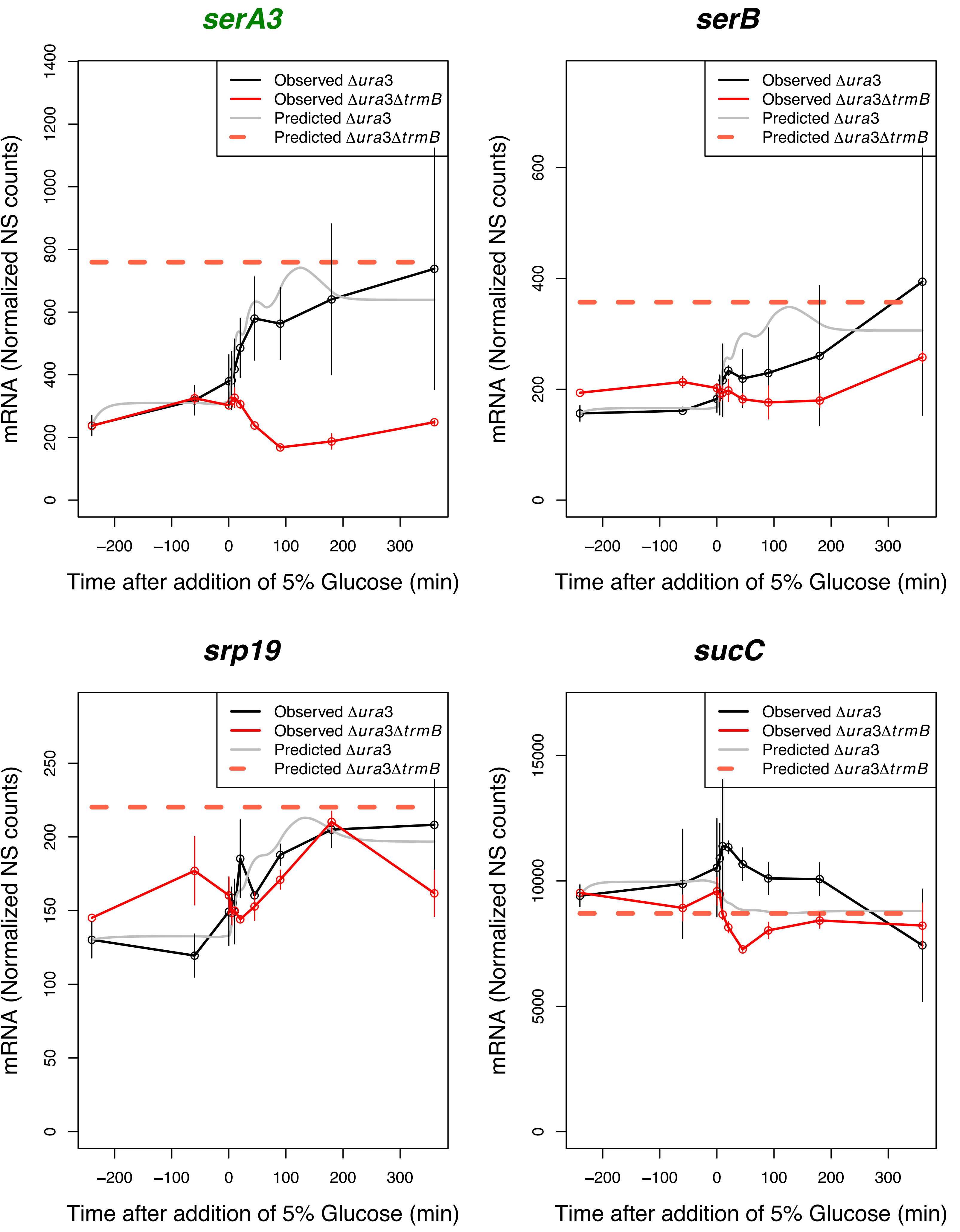
serA3
serB
srp19
sucC
Observed Δura3
Observed Δura3ΔtrmB
Predicted Δura3
Predicted Δura3ΔtrmB
mRNA (Normalized NS counts)
Time after addition of 5% Glucose (min)
−200
−100
0
100
200
300
0
200
400
600
800
1000
1200
1400
50
150
250
5000
10000
15000

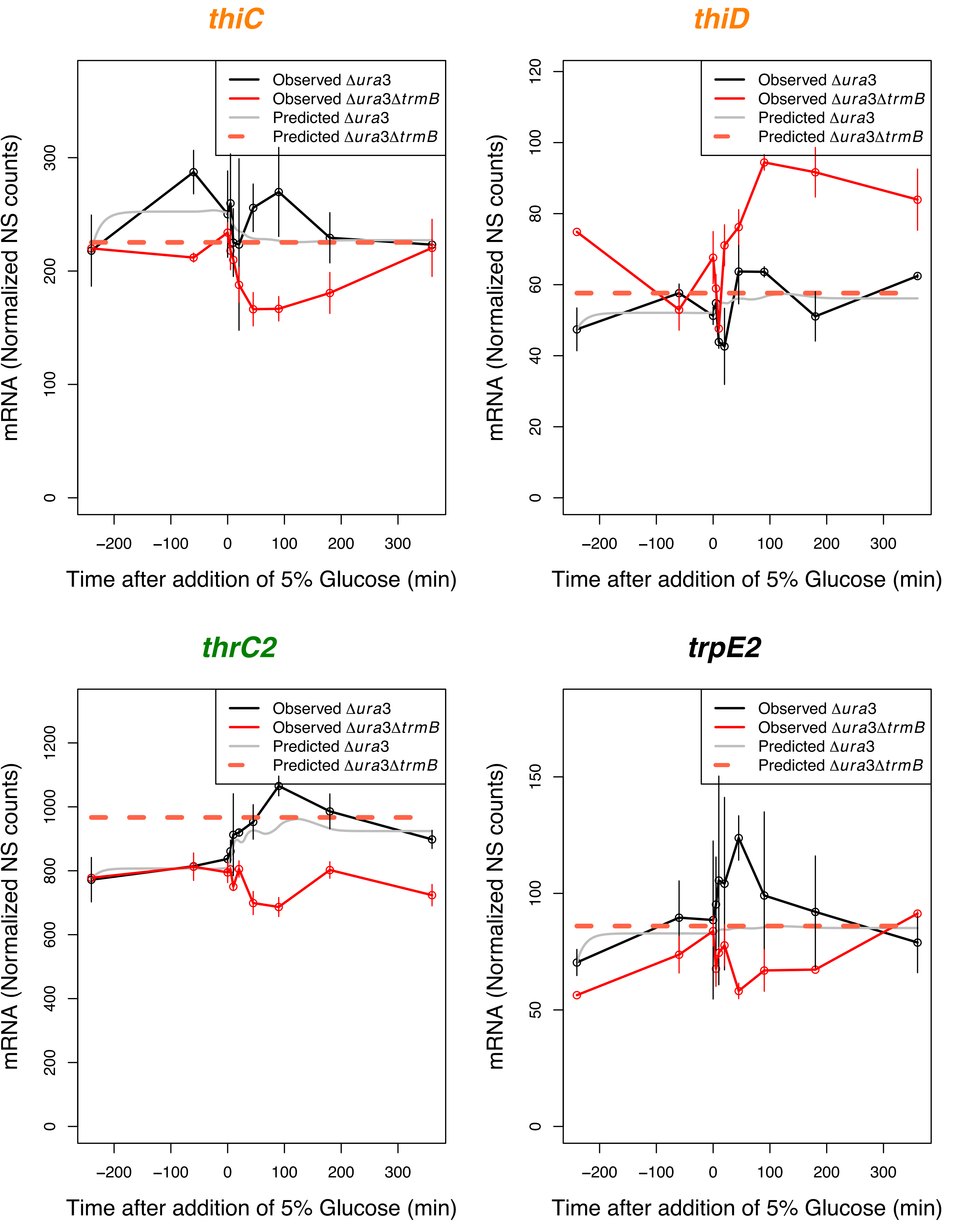
thiC
thiD
thrC2
trpE2
Observed Δura3
Observed Δura3ΔtrmB
Predicted Δura3
Predicted Δura3ΔtrmB
mRNA (Normalized NS counts)
Time after addition of 5% Glucose (min)
−200
−100
0
100
200
300
20
40
60
80
100
120
400
600
800
1000
1200
50
150

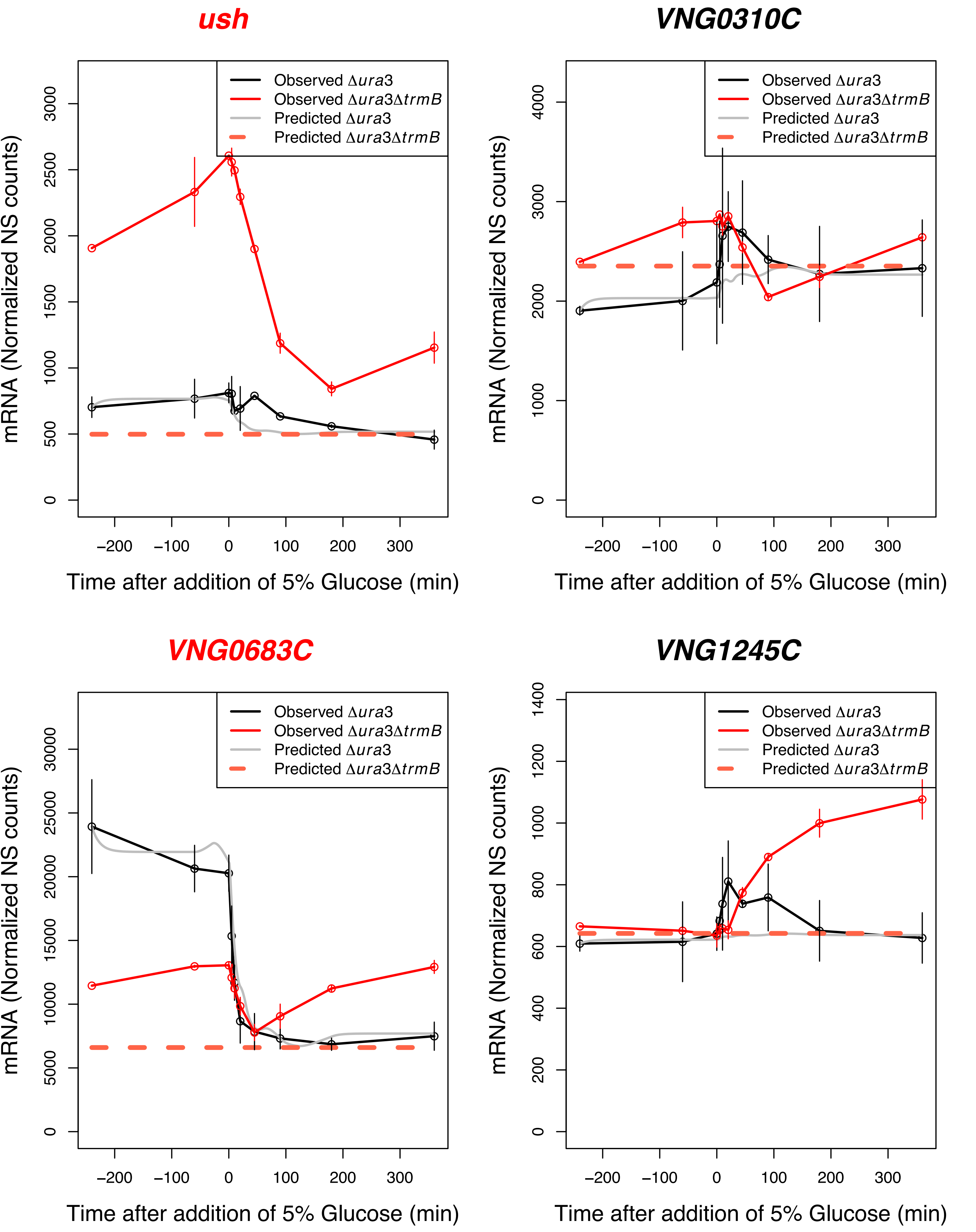
ush
VNG0310C
VNG0683C
VNG1245C
Observed Δura3
Observed Δura3ΔtrmB
Predicted Δura3
Predicted Δura3ΔtrmB
mRNA (Normalized NS counts)
Time after addition of 5% Glucose (min)
−200
−100
0
100
200
300
0
500
1000
1500
2000
2500
3000
4000
5000
10000
15000
20000
25000
30000
200
400
600
800
1200
1400

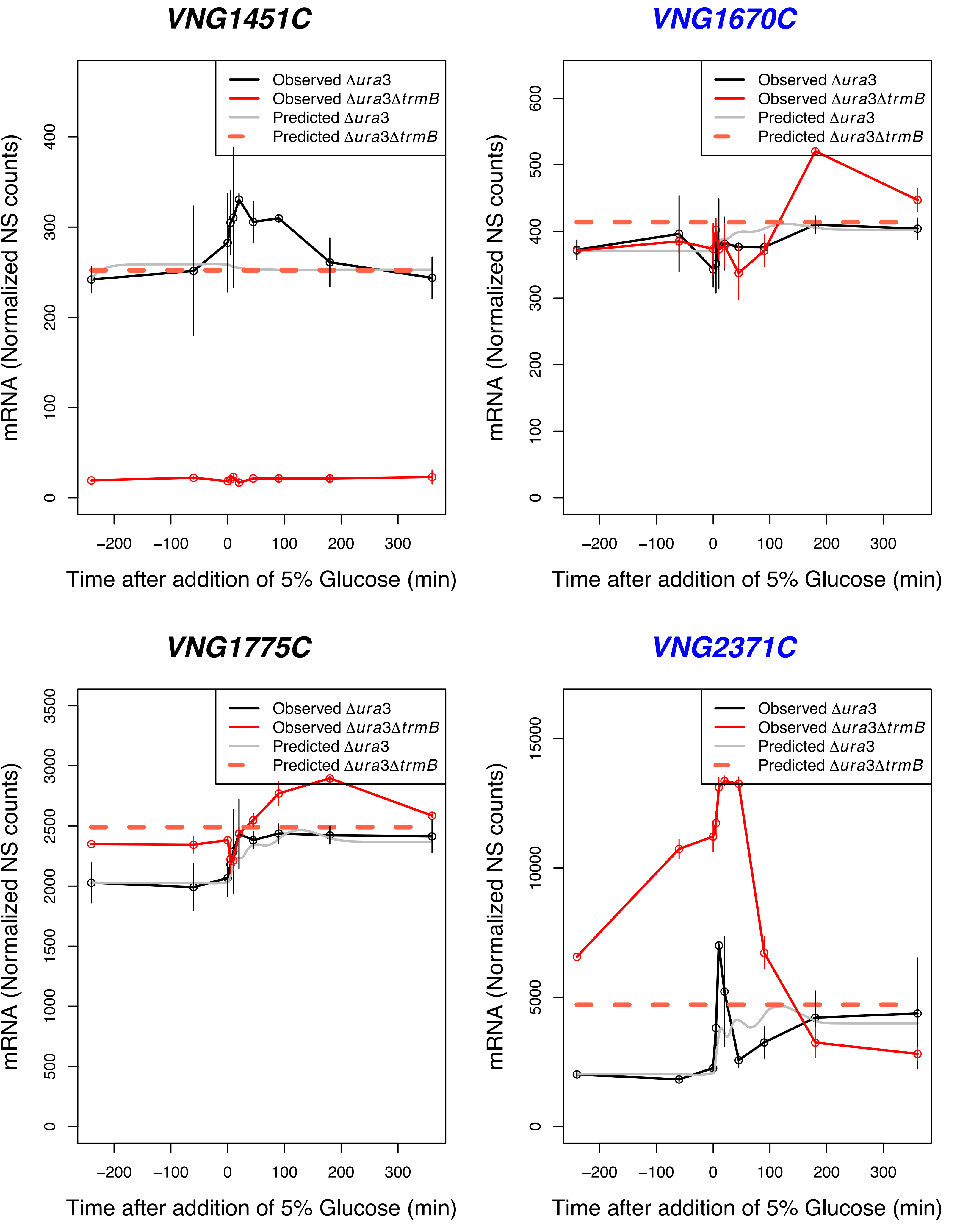
VNG1451C
VNG1670C
VNG1775C
VNG2371C
Observed Δura3
Observed Δura3ΔtrmB
Predicted Δura3
Predicted Δura3ΔtrmB
mRNA (Normalized NS counts)
Time after addition of 5% Glucose (min)
−200
−100
0
100
200
300
400
500
600
1000
1500
2000
2500
3000
3500
5000
10000
15000

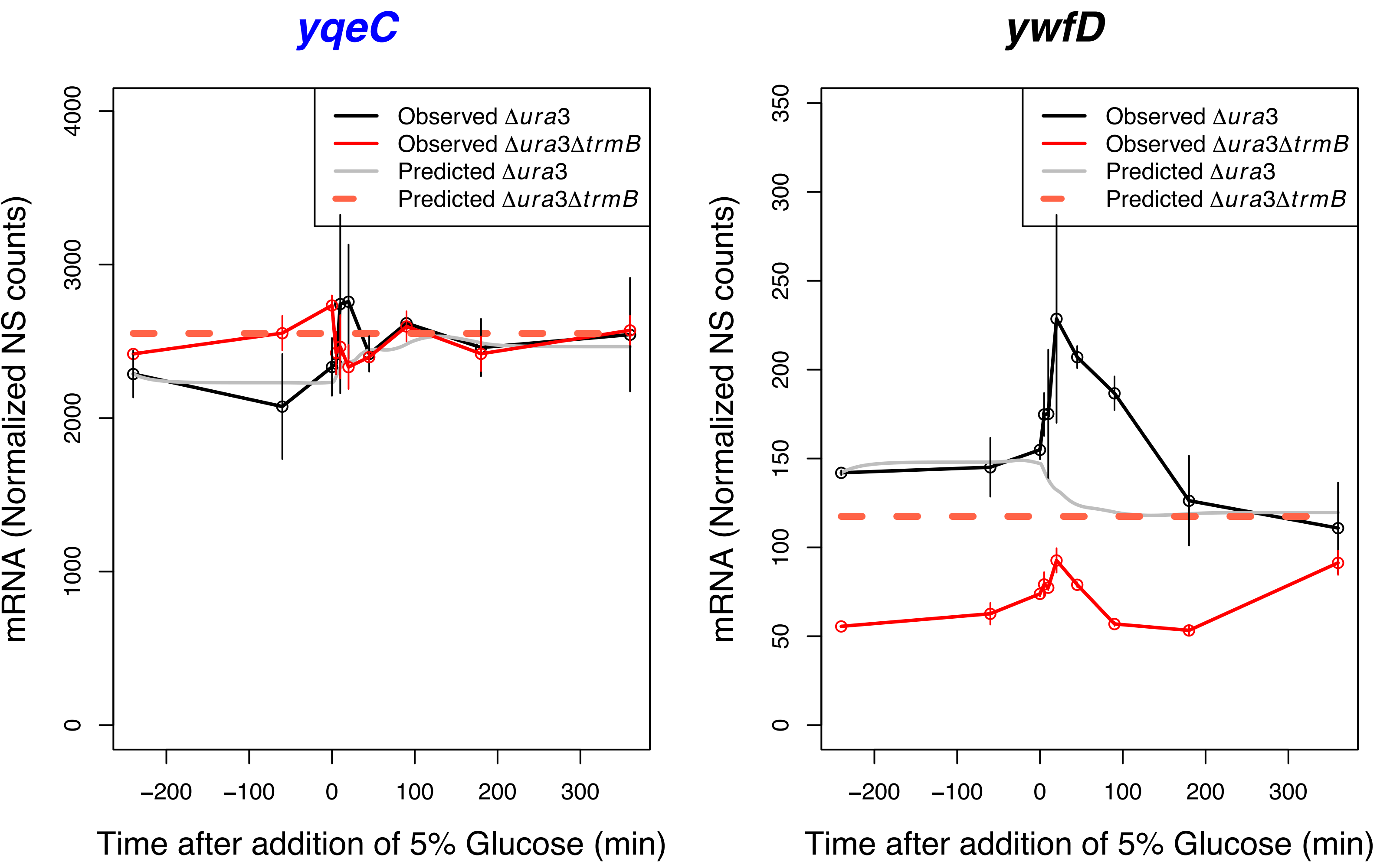
yqeC
ywfD
Observed Δura3
Observed Δura3ΔtrmB
Predicted Δura3
Predicted Δura3ΔtrmB
mRNA (Normalized NS counts)
Time after addition of 5% Glucose (min)
0
1000
2000
3000
4000
0
50
100
150
200
250
300
350
−200
−100
0
100
200
300
