# Supplementary material for: Protein–DNA binding dynamics predict transcriptional response to nutrients in archaea
Source: Nucleic Acids Res. 2013 Jul 26;41(18):8546–58. doi: 10.1093/nar/gkt659 (PMC3794607; doi:10.1093/nar/gkt659)
Supplement: Supplementary Data [file supp_gkt659_nar-01494-h-2013-File003.pdf]

Supplementary Figure 2

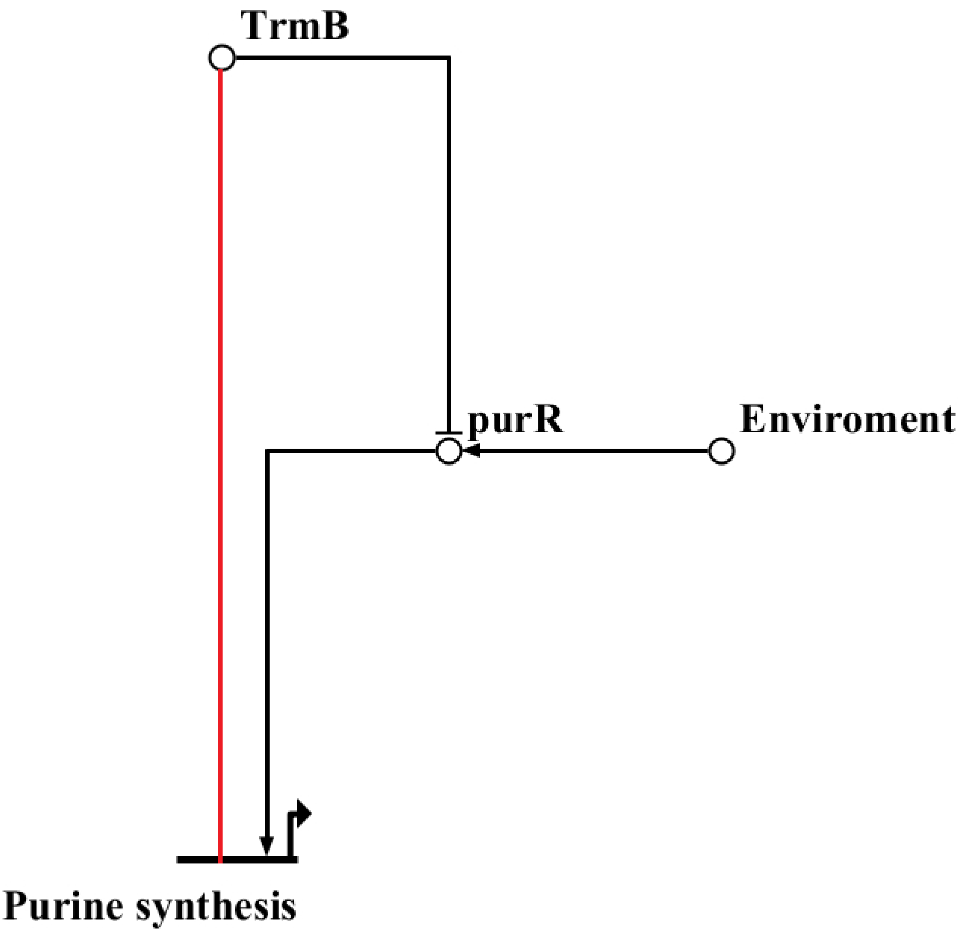

Supplementary Figure 3. PurR is the primary regulator of purine biosynthesis. Its level is regulated by TrmB. TrmB may weakly control purine synthesis genes as well. Model of proposed regulation for purine biosynthesis. PurR refers to a putative regulator. The red line indicates weak, potentially direct regulation.

Supplementary Figure 3

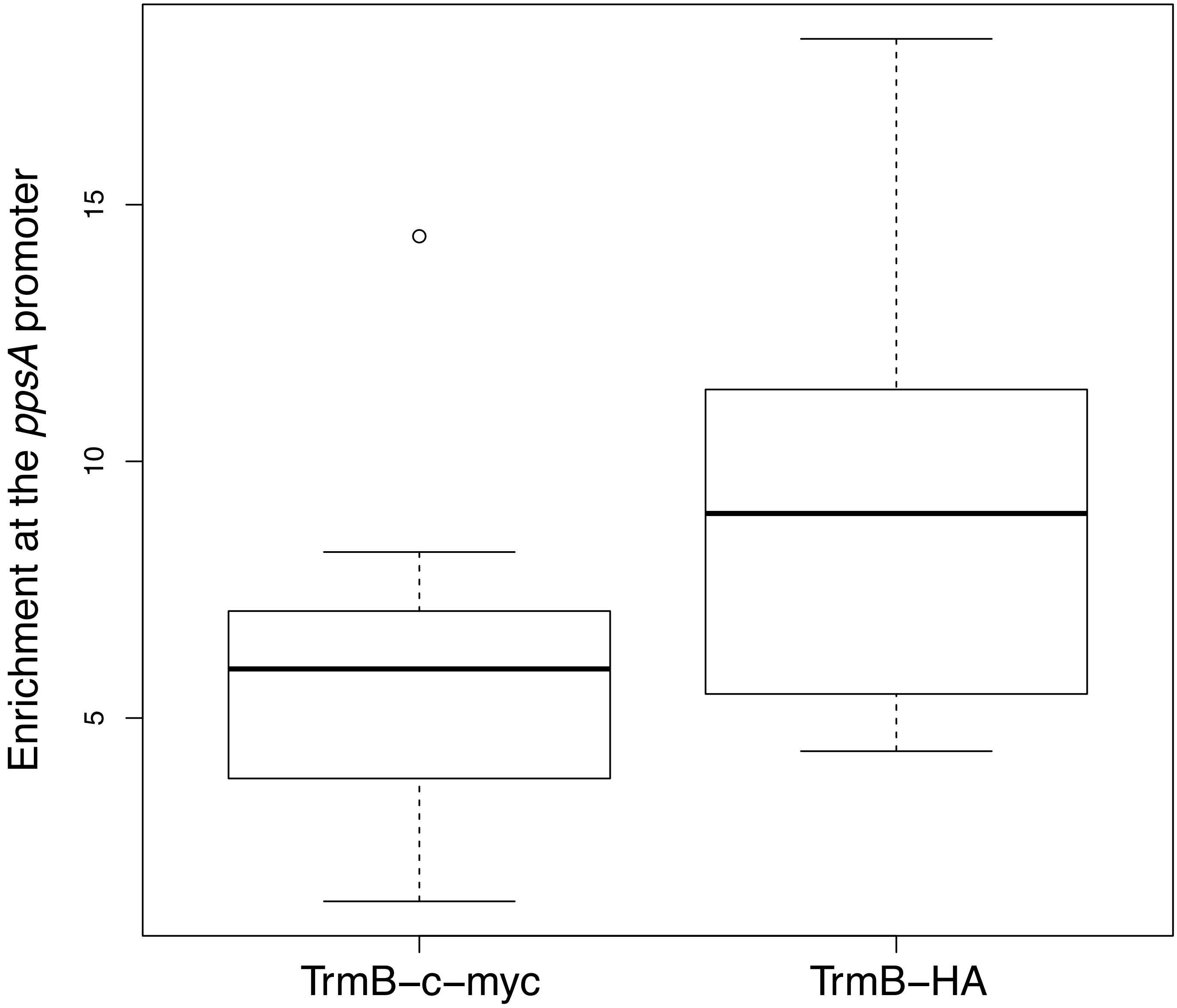

Supplementary Figure 3. Boxplot showing the relative enrichment of TrmB at the *ppsA* promoter in the absence of glucose using ChIP-qPCR. The first box is a TrmB-c-myc fusion expressed from a plasmid using the ferredoxin promoter. The second box is a chromosomally integrated TrmB-HA fusion being expressed from the native promoter. The two-sided unequal varience t-test returns a *p*-value of 0.175, suggesting that there is no significant difference between the two.
