# Supplementary material for: Protein–DNA binding dynamics predict transcriptional response to nutrients in archaea
Source: Nucleic Acids Res. 2013 Jul 26;41(18):8546–58. doi: 10.1093/nar/gkt659 (PMC3794607; doi:10.1093/nar/gkt659)
Supplement: Supplementary Data [file supp_gkt659_nar-01494-h-2013-File004.docx]

**SUPPLEMENTARY TABLES**

Supplementary Table 1 - Strains used

| **Strain** | **Reference** | **Notes** |
| --- | --- | --- |
| *∆ura3* | (Peck *et al*, 2000) | In frame deletion of *VNG1673G* (*ura3*). Counterselectable marker. |
| *∆ura3∆trmB* | (Schmid *et al*, 2009) | In frame deletion of VNG1451C. |
| *trmB::c-myc* | (Schmid *et al*, 2009) | Constitutively expressed c-myc tagged TrmB. |

Supplementary Table 2 - CDM Media – Adapted from (Schmid *et al*, 2009)

| **Component** | **Amount (mM)** |
| --- | --- |
| NaCl | 4278.0 |
| MgSO_4_·7H2O | 40.000 |
| MOPS | 40.000 |
| KCl | 27.000 |
| NaH_2_PO_4_ | 0.1670 |
| Folic Acid | 0.0113 |
| Thiamine HCl | 0.0148 |
| Biotin | 0.0205 |
| L-ala | 5.7100 |
| L-arg-HCl | 1.9000 |
| L-asn | 0.9600 |
| L-asp | 1.8800 |
| L-glu | 10.640 |
| L-gln | 5.0000 |
| L-gly | 2.0000 |
| L-his-HCl | 0.2390 |
| L-ile | 3.3500 |
| L-leu | 6.1000 |
| L-lys-HCl | 1.0400 |
| L-met | 0.6030 |
| L-phe | 0.3030 |
| L-pro | 0.4000 |
| L-ser | 2.9020 |
| L-thre | 4.2000 |
| L-trp | 0.0980 |
| L-tyr | 0.6180 |
| L-val | 2.1350 |
| MnSO_4_ | 0.0100 |
| FeSO_4_ | 0.0100 |
| ZnSO_4_ | 1.0E-05 |
| CuSO_4_ | 1.0E-05 |

Supplementary Table 3 - Primer sequences

| **Primer** | **Sequence** | **Purpose** |
| --- | --- | --- |
| *pykA* fwd | CAAGCCGCTTGCGGTGATG | RT-qPCR |
| *pykA* rev | CGCTGCCGGTTTCGATGTG | RT-qPCR |
| *ppsA* fwd | TGGGCGAGCCTGTTCAC | RT-qPCR |
| *ppsA* rev | CGACGGCGATGTTCACG | RT-qPCR |
| *VNG1756G* Fwd | ACGAAGTGTTTGCGGTCG | RT-qPCR |
| *VNG1756G* Rev | TCCACGTCCGGTATTTCATG | RT-qPCR |
| *ppsA*_BP_fwd | TCTTCCAGCCAGCGTACAG | 5' end of *ppsA* for ChIP-qPCR |
| *ppsA*_BP_rev | CGGTTCCGAGTACCATATGTC | 5' end of *ppsA* for ChIP-qPCR |
| *ppsA*_3'_fwd | CCGCGCCTCCCTAATCCC | 3' end of *ppsA* for ChIP-qPCR |
| *ppsA*_3'_rev | GCGTCGAGCAGCGACTCC | 3' end of *ppsA* for ChIP-qPCR |

Supplementary Table 4 - NanoString probe identities and sequences used in this study (see additional supplementary XLS file).

Supplementary Table 5 - Calculation of degradation constants from (Hundt *et al*, 2007); see separate supplementary CSV file.

Supplementary Table 6 - All raw and normalized NanoString gene expression data across 10 points of the glucose response time course (see separate supplementary XLS file).

Supplementary Table 7 - Abbreviations used in Figure 4.

| **Abbreviation** | **metabolite/enzyme** |
| --- | --- |
| 1-(5P-ribosyl) N-FGAM | 1-(5'-phosphoribosyl)-N-formylglycinamidine |
| 1,3-DPG | 1,3-diphosphoglycerate |
| 2-PG | 2-phosphoglycerate |
| 3DHQ | 3-dehydroquinate |
| 3-DHS | 3-dehydroshikimate |
| 3-EPS-5-P | 3-enolpyruvylshikimate-5-phosphate |
| 3HP | 3-phospho-hydroxypyruvate |
| 3-PG | 3-phosphoglycerate |
| ADTH | 2-amino-3,7-dideoxy-D-threo-hept-6-ulosonate |
| AICAR | 5-phosphoribosyl-4-carboxamide-5-aminoimidazole |
| AIR | 5-phosphoribosyl-5-aminoimidazole |
| aKG | α-ketoglutarate |
| DKFP | 6-deoxy-5-ketofructose-1-phosphate |
| F1,6P | Fructose-1,6-bisphosphate |
| F6P | Fructose-6-phosphate |
| FAICAR | 5-phosphoribosyl-4-carboxamide-5-formylaminoimidazole |
| FGAM | 5-phosphoribosyl-N-formylglycinamidine |
| G3P | Glyceraldehyde-3-phosphate |
| G6P | Glucose-6-phosphate |
| GAR | 5-phosphoribosylglycinamide |
| HMP | 4-Amino-5-hydroxymethyl-2-methylpyrimidine |
| HMP-P | 4-Amino-2-methyl-5-phosphomethylpyrimidine |
| IMP | Inosine monophosphate |
| KDG | 2-keto-3-deoxygluconate |
| KDPG | 2-keto-3-deoxy-6-phosphogluconate |
| MG | Methylglyoxal |
| NCAIR | 5-phosphoribosyl-5-aminoimidazole-4-carboxylic acid |
| PEP | Phosphoenolpyruvate |
| PRPP | 5-phospho-D-ribosyl-1-pyrophosphate |
| SAICAR | Phospho-ribosylaminoimidazole-succinocarboxamide |
